# Nascent chains can form co-translational folding intermediates that promote post-translational folding outcomes in a disease-causing protein

**DOI:** 10.1038/s41467-021-26531-1

**Published:** 2021-11-08

**Authors:** Elena Plessa, Lien P. Chu, Sammy H. S. Chan, Oliver L. Thomas, Anaïs M. E. Cassaignau, Christopher A. Waudby, John Christodoulou, Lisa D. Cabrita

**Affiliations:** 1 Institute of Structural and Molecular Biology, University College London, Gower Street, London, WC1E 6BT UK; 2 School of Crystallography, Birkbeck College, University of London, Malet Street, London, WC1E 7HX UK

**Keywords:** Protein aggregation, Ribosome, Solution-state NMR

## Abstract

During biosynthesis, proteins can begin folding co-translationally to acquire their biologically-active structures. Folding, however, is an imperfect process and in many cases misfolding results in disease. Less is understood of how misfolding begins during biosynthesis. The human protein, alpha-1-antitrypsin (AAT) folds under kinetic control via a folding intermediate; its pathological variants readily form self-associated polymers at the site of synthesis, leading to alpha-1-antitrypsin deficiency. We observe that AAT nascent polypeptides stall during their biosynthesis, resulting in full-length nascent chains that remain bound to ribosome, forming a persistent ribosome-nascent chain complex (RNC) prior to release. We analyse the structure of these RNCs, which reveals compacted, partially-folded co-translational folding intermediates possessing molten-globule characteristics. We find that the highly-polymerogenic mutant, Z AAT, forms a distinct co-translational folding intermediate relative to wild-type. Its very modest structural differences suggests that the ribosome uniquely tempers the impact of deleterious mutations during nascent chain emergence. Following nascent chain release however, these co-translational folding intermediates guide post-translational folding outcomes thus suggesting that Z’s misfolding is initiated from co-translational structure. Our findings demonstrate that co-translational folding intermediates drive how some proteins fold under kinetic control, and may thus also serve as tractable therapeutic targets for human disease.

## Abstract (web summary)

Alpha-1-antitrypsin (AAT) deficiency results from misfolding-prone AAT variants. Here the authors show that AAT forms co-translational folding intermediates on the ribosome that persist upon release and determine its folding fate. They show too that the ribosome can also modulate misfolding-prone AAT intermediates during their synthesis.

## Introduction

Within living systems, a crucial housekeeping network is in place to maintain cellular proteostasis^1^, with severe imbalances being linked to the onset of many common human diseases^2^. A central process within this network is the necessity for the vast majority of newly-synthesised polypeptide chains to fold and acquire their biologically-active, three-dimensional structures. It is well-established that during biosynthesis, protein folding can begin co-translationally on the ribosome to promote efficient folding outcomes^3^. Protein folding, however, is also known to compete with misfolding^4^ and evidence from cellular studies in which actively-translating nascent chains can be ubiquitinated and targeted for degradation^5–7^ suggests that aberrant folding processes begin during biosynthesis. In vitro studies using molecular tweezers have shown that multi-domain proteins are capable of misfolding on the ribosome^8,9^, however a detailed molecular understanding of co-translational misfolding remains to be explored.

**Fig. 1:**
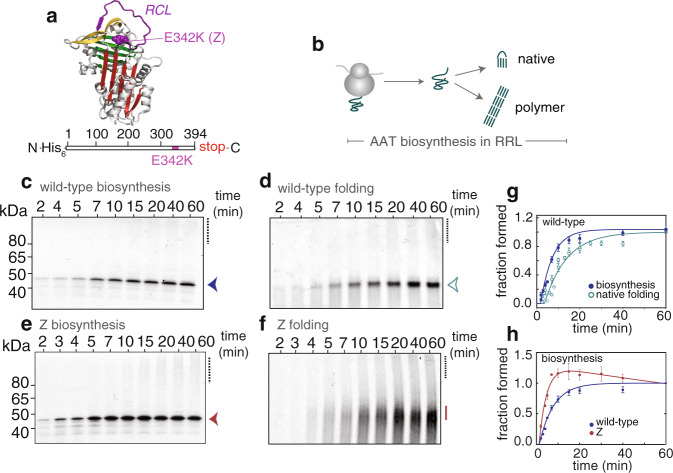
Monitoring the folding and misfolding of alpha-1-antitrypsin (AAT) during biosynthesis in rabbit reticulocyte lysate (RRL) using ^35^S methionine radiolabelling. **a** (upper) AAT structure (PDB ID: 1QLP) with landmarks highlighted: A-sheet (red), B-sheet (green), C-sheet (yellow), reactive centre loop (RCL, purple), and site of the Z mutation (E342K). (lower*)* Schematic of the full-length AAT DNA construct and highlighting the relative position of the Z (E342K) mutation. **b** Scheme of released AAT biosynthesis and folding in RRL. **c** Production of wild-type AAT nascent chains as followed by denaturing PAGE. Highlighted are the full-length species (arrow), and high molecular weight species / polymerisation (i.e., a smear) as a dashed line. **d** Folding of wild-type AAT nascent chains as monitored by native PAGE (using the same samples as in **c**). Highlighted is the biologically-active, monomeric species (arrow) and polymer (dashed line). **e** Production of Z nascent chains as followed by denaturing PAGE. Highlighted are the full-length species (arrow), and high molecular weight species / polymerisation (i.e., a smear) as a dashed line. **f** Folding of Z AAT nascent chains as monitored by native PAGE (using the same samples as in **e**). Unlike wild-type in **c**, Z nascent chains cannot fold efficiently (solid line) and form polymers (dashed line). **g** Biosynthesis and native folding of wild-type AAT nascent chains (analysis from **c**, **d**; *n* = 4). **h** Biosynthesis of wild-type and Z AAT nascent chains over 60 min. Data have been normalised to the reaction end-point to enable a comparative analysis. All samples were flash-frozen and treated with RNase A prior to analysis. (*n* = 5 biological repeats). Data are presented as mean values +/− SEM.

The human glycoprotein, alpha-1-antitrypsin (AAT) is a serine protease inhibitor (serpin)^10^ and whose misfolding is implicated in the human disease, alpha-1-antitrypsin deficiency (AATD). AAT is synthesised and folds within the endoplasmic reticulum of hepatocytes and is secreted into the bloodstream where it acts predominantly as an inhibitor of neutrophil elastase in the lungs^11^. The mature protein’s metastable native fold comprises a single-domain topology composed of three beta sheets, nine alpha helices and a solvent-exposed reactive-centre loop (Fig. 1a). This metastability imparts dynamic properties necessary for AAT’s inhibitory function, while point mutations render AAT vulnerable to misfolding and self-assembly, causing the formation of polymers at the site of synthesis^12^. One such point mutation, E342K, denoted as “Z”, contributes to extensive polymerisation and aggregation in hepatocytes, resulting in a subsequent loss of AAT within the bloodstream, and is responsible for the most severe form of AATD^11^. Biophysical studies of AAT show that it folds slowly (minutes time-scale) via a multi-state pathway forming at least one folding intermediate^13–15^. Comparative biophysical studies of wild-type (“M”) and Z AAT show that their native states are thermodynamically indistinguishable, however biochemical and biophysical studies have shown that Z readily forms a long-lived, kinetically-trapped intermediate during folding^13–15^, which has been characterised at equilibrium using NMR spectroscopy^16^. These studies, when combined, reveal that the origin of Z AAT’s polymerisation lies in a “kinetic folding defect”^13^.

We have investigated the origin of this kinetic folding defect by systematically studying the folding and misfolding properties of newly synthesising AAT polypeptide chains as they emerge and are released from the ribosome machinery. Here we show that full-length AAT nascent chains stall at their C-termini on ribosomes and persist; this enables structure to begin forming co-translationally. Using PEGylation and NMR spectroscopy we provide structural models of the ribosome-bound wild-type and Z nascent chains, which reveal that they form distinct co-translational folding intermediates possessing molten-globule characteristics and compacted N-termini. We find that there are small structural differences between ribosome-bound wild-type and Z nascent chains, suggestive of a tempering effect imposed by the ribosome. Following the release of the nascent chain, however, these co-translational folding differences persist and are exacerbated post-translationally to influence AAT’s folding fate. In this work, we thus show a basis of a folding-misfolding branchpoint that is initiated on the ribosome, and is a finding which has implications for targeting conformational diseases.

## Results

### A cell-free system shows that wild-type AAT completes its native fold post-translationally during biosynthesis whilst Z AAT remains kinetically trapped as partially-folded species

To evaluate the relationship between folding and misfolding as it occurs during biosynthesis, we studied the production of hexahistidine-tagged, wild-type and Z AAT using a coupled transcription–translation, rabbit reticulocyte lysate (RRL) cell-free system (Fig. 1a). Since AAT can acquire its native, biologically-active structure in the absence of glycosylation^17^, we measured folding outcomes of nascent polypeptide chains (NC) during biosynthesis in the absence of microsomal membranes in order to maximise protein yields (Fig. 1b). The biosynthesis reactions were also synchronised with the translational initiation inhibitor, aurintricarboxylic acid^18,19^, in order to limit the ribosomes to a single round of translation. As monitored using ^35^S-methionine radiolabelling, full-length, wild-type AAT was synthesised within 5 min (rate of full-length AAT production, 0.18 ± 0.02 min^−1^) following an initial lag time of ~ 2 min, (Fig. 1c, g and Supplementary Fig. 1a). The appearance of natively-folded AAT could be monitored by native PAGE, and was only detected after ~6 min (rate of full-length AAT folding, 0.096 ± 0.012 min^−1^) as observed by the presence of a discrete band^20,21^ (Fig. 1d, g and Supplementary Fig. 1a, b). Overall, these data show that AAT folds slowly during biosynthesis and its native structure is completed post-translationally.

Z nascent chains are generated within a similar time frame as those of wild-type and where the fraction of the full-length species produced, slightly decreased over time (Fig. 1e, h and Supplementary Fig. 1a). These results suggest that the apparently faster rate of production of Z reflects an additional process occurring during biosynthesis. Unlike for wild-type, Z nascent chains did not fold readily within the 60 min reaction time-frame. This is shown as a diffuse monomeric banding pattern on native PAGE (Fig. 1f), which is characteristic of the formation of a non-native species associated with Z AAT’s folding defect^13^. Also present during Z’s biosynthesis was the concomitant formation of a high-molecular weight species shown as a smear across much of the upper region in the gel, consistent with self-association and polymerisation observed for AAT in vitro^20^ (Fig. 1e, f). Polymerisation was similarly observed for wild-type AAT (Fig. 1c, d), but to a lesser extent relative to Z. Overall, these data illustrate that an inherent competition between folding and misfolding processes begins at the earliest stages of nascent chain biosynthesis.

### Translating ribosomes form persistent, full-length AAT RNCs during biosynthesis

**Fig. 2:**
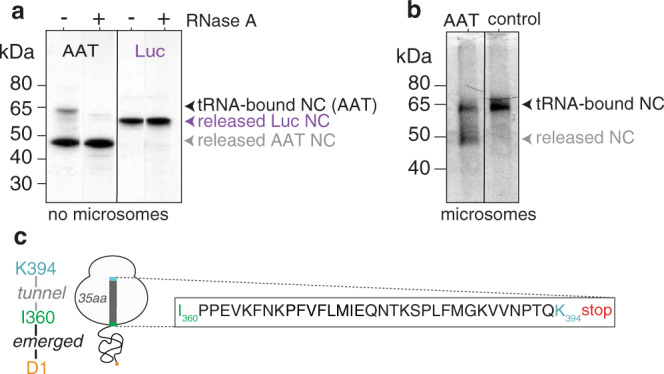
AAT forms persistent full-length ribosome-nascent chain complexes. **a** Biosynthesis of AAT and luciferase (Luc) after a 60 min biosynthesis reaction quenched with cycloheximide in the absence of microsomes (both DNA constructs contain stop codons). Highlighted are the full-length, tRNA-bound nascent chains (NC) and full-length, released nascent chains. **b** Biosynthesis of AAT in the presence of microsomes, alongside a control (AAT DNA construct with no stop codon to force production of RNCs), highlighting that both full-length tRNA-bound nascent chains (NC) and released nascent chains can be formed in the endoplasmic reticulum. Source data are provided as a Source Data file. **c** Schematic of a full-length AAT RNC showing the sequence expected within the tunnel and beyond the ribosome. Note that **a** and **b** show ^35^S methionine detection via partially-denaturing PAGE (see “Methods” section).

To investigate whether misfolding can occur during biosynthesis on the ribosome, we adapted the synchronised experiments in rabbit reticulocyte lysate described above, and terminated the reactions with cycloheximide, rather than RNase A. Cycloheximide binds at the E-site of the 80S ribosome’s peptidyl transferase centre^22^ and halts elongation during translation, and is used here to retain any long-lived, intact ribosome-nascent chain complexes (RNC; nascent chains are bound to the ribosome via an ester linkage to a P-site tRNA moiety). A 60 min biosynthesis reaction of wild-type AAT, revealed intact, full-length RNCs (~64 kDa), despite the presence of a stop codon within the DNA construct (Fig. 2a). These RNCs unexpectedly made up a substantial population (~27% of total species) in the reaction relative to full-length, released AAT nascent chains (Fig. 2a and Supplementary Fig. 1c, d). RNC formation was, however, not observed for firefly luciferase (Fig. 2a), suggesting that this is a feature specific to AAT. We also targeted AAT (containing its natural signal sequence) to canine pancreatic microsomes (an endoplasmic reticulum analogue), and evaluated its biosynthesis following a 60 min reaction. Following purification of the microsomes using sucrose flotation^23^, AAT was found to have expressed both as full-length RNCs, as well as full-length, released nascent chains (Fig. 2b and Supplementary Fig. 1e). These data thus suggest that translational arrest is inherent to this protein; an inspection of AAT’s C-terminal 35 amino acids (P360-K394) which are sequestered within the tunnel, however, shows no discerning motifs (e.g., polyproline^24^ or polybasic^25^ amino acids) that are typically associated with ribosome translational arrest (Fig. 2c).

We then examined how these full-length AAT RNCs formed over time during biosynthesis and found that they are a relatively long-lived species, peaking at ~ 20 min. This formation of persistent, full-length AAT RNCs may likely relate to the protein’s need for translocation, N-linked glycosylation and folding in the endoplasmic reticulum^26^, the location where AAT is typically produced within the cell.

### Full-length AAT RNCs are capable of forming structure

**Fig. 3:**
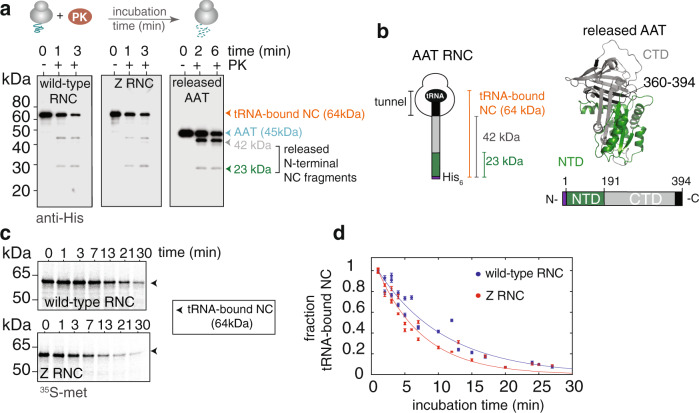
Wild-type and Z AAT ribosome-nascent chain complexes (RNC) can adopt co-translational structure. **a** A schematic of the proteinase K limited proteolysis experiment (upper). Limited proteolysis of wild-type and Z purified RNCs (orange arrowhead), and purified, released wild-type AAT (blue arrowhead) monitored by an anti-His western blot (lower). Highlighted are two N-terminal proteolytic products released from the nascent chain segment emerged from the ribosome: 42 kDa (grey arrowhead) and 23 kDa (green arrowhead). **b** Schematic of an AAT RNC highlighting the N-terminal fragments observed in **a** (left). (right) Structure of AAT and schematic representation of (released) AAT showing the boundaries of the N-terminal (NTD, green) and C-terminal (CTD, grey) sub-domains, and C-terminal residues 360–394 which are occluded in the ribosomal tunnel within the RNC (see left). The segment of structure shown in black is the 35 amino acid stretch that is occluded in the tunnel when full-length AAT is bound to the ribosome. **c** Proteinase K limited proteolysis measured over time for wild-type and Z RNCs, highlighting the measurement of the intact tRNA-bound nascent chain (NC) over time using ^35^S-methionine detection and partially-denaturing PAGE (*n* = 3 biological repeats). See also Supplementary Fig. 2. **d** Densitometric analysis of the proteinase K time-course from **c** with exponential fits. Data are presented as mean values +/− SEM.

During expression in the endoplasmic reticulum, any full-length AAT RNCs that form at any given time are expected to have ~30–35 C-terminal amino acids sequestered in the ribosomal exit tunnel^27^ and a further 20 amino acids by the translocation machinery and membrane^28^. We examined AAT RNCs in the absence of microsomes as a simplified, representative model for the folding processes possible within the endoplasmic reticulum. In these full-length AAT RNCs, amino acids 1–360 (corresponding to 90% of the completely translated sequence, 394 aa) is expected to have emerged from the ribosomal exit tunnel; the remainder would be within the ribosome tunnel (Fig. 2c). We performed limited proteolysis experiments using proteinase K on purified wild-type RNCs and analysed the released fragments^29^ via partially-denaturing PAGE. As detected by ^35^S-methionine radiolabelling, a collection of protease-resistant AAT fragments was produced over time (Supplementary Fig. 2a–c), suggesting that the segments that have emerged from the tunnel in these bound nascent chains are capable of forming structure. Next, by exploiting the nascent chain’s N-terminal His-tag, we used an anti-His western blot to evaluate which of the observed fragments had originated from the tRNA-bound nascent chain (Fig. 3a and Supplementary Fig. 2a). This experiment revealed two prominent N-terminal fragments of *~* 42 and *~*23 kDa, which were also similarly observed in released AAT (Fig. 3a). These data demonstrate that the ribosome-bound nascent chains are capable of adopting persistent, compacted structure within the tunnel-emerged segment (D1-I360; 35 aa buried in the tunnel) (Supplementary Fig. 2d, e). The equivalent proteolysis experiments were performed on Z RNCs (Fig. 3a), which produced fragments of similar sizes to those of wild-type RNC, suggesting that when bound to the ribosome, both wild-type and Z nascent chains likely form similar structures.

To identify the sequence of the fragments released from both the wild-type and Z RNCs, mass spectrometry analysis was performed on an equivalent sample of released, wild-type AAT. These data showed that the N-terminal fragments that were observed in the RNC experiments were 42.2 kDa (1–365 aa) and 22.8 kDa (1–191 aa) in size (Supplementary Fig. 2f–h). These data show that the ribosome-bound, full-length AAT nascent chain thus forms two sub-domains, with boundaries consistent with predictions made for the released natively-folded protein^30^: a compact N-terminal fragment (D1-K191) and a more labile C-terminal fragment (G192-K394; residues P361-K394 are buried in the tunnel), where the relative compactness was characterised using limited proteolysis (Fig. 3b and Supplementary Fig. 2b, c, e).

We also assessed using limited proteolysis whether we could detect any differences in the structures formed by the wild-type and Z RNCs. We monitored the integrity of the RNC (i.e., the tRNA-bound nascent chain) over time and found that Z RNCs degraded ~1.5 times faster than wild-type, with rates of 0.74 ± 0.04 and 0.50 ± 0.03 min^−1^, respectively (Fig. 3c, d). This small but discernible difference in rate suggests that the global structures formed by wild-type and Z RNCs differ marginally in stability.

### Full-length AAT ribosome-nascent chain complexes possess a compacted N-terminus

**Fig. 4:**
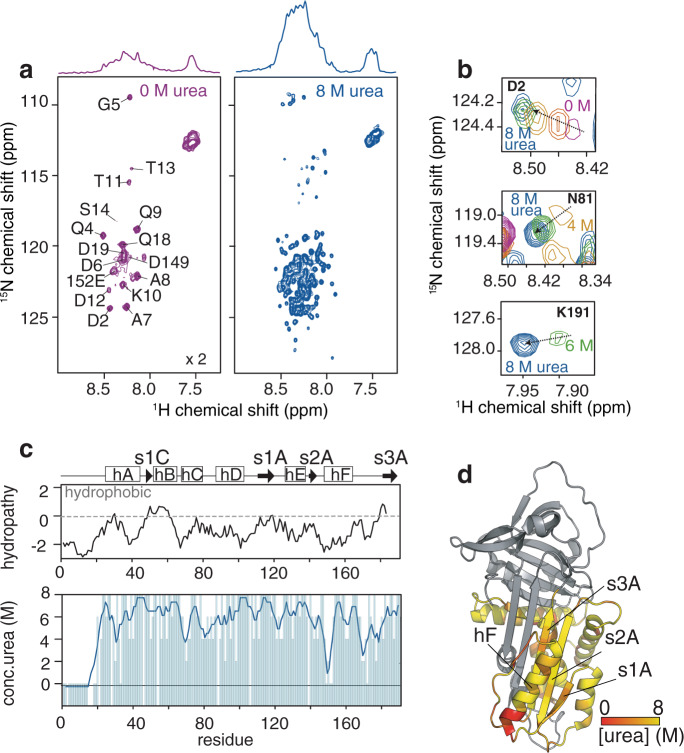
NMR characterisation of the N-terminal fragment AAT-191. **a** (upper) 1D and (lower) 2D ^1^H,^15^N-SOFAST HMQC spectra of the AAT-191 fragment in (left) 0 M and (right) 8 M urea, recorded at 25 °C and at a ^1^H frequency of 950 MHz. **b** Selected AAT-191 resonances observed in increasing concentrations of urea. **c** (upper) A Kyte and Doolittle hydropathy plot for AAT-191 (lower) A plot depicting the urea concentrations at which non-proline residues are first observable, and includes a 3-point moving average to highlight the extent of compaction observed across the sequence. **d** Mapped onto the AAT structure is a representation of AAT-191 depicting the regions that progressively unfold in 8 M urea and at which stage NMR resonances first become observable (**c**).

To examine further the minimal polypeptide sequence capable of forming structure co-translationally in the AAT RNCs, we generated the 23 kDa N-terminal fragment (AAT-191) identified by limited proteolysis in *E.coli* (Fig. 3a, b and Supplementary Fig. 3a) and used NMR spectroscopy to characterise its structural properties at high-resolution (Fig. 4a–d). We used ^1^H,^15^N-correlation spectra, which report on the backbone of a polypeptide chain, to provide a residue-specific “fingerprint” of its structural and dynamic properties (Fig. 4a, b). The resulting spectra of released AAT-191 revealed only 18 intense resonances (of the 185 possible non-proline resonances) which were narrowly-dispersed within the ^1^H chemical shift, which is a feature consistent with the presence of disordered regions (Fig. 4a and Supplementary Fig. 3b). The absence of observable resonances for the majority of AAT’s residues indicates that they undergo substantial line broadening and thus appear to provide no observable signals (Fig. 4a, c). Translational diffusion measurements, however, confirmed that AAT-191 was monomeric, indicating that the line broadening did not relate to protein aggregation (Fig. S3c). Under denaturing conditions in 8 M urea, all of the expected non-proline resonances could be resolved (Fig. 4a and Supplementary Fig. 3b). A urea titration approach was then used to assign the native spectra (Fig. S3b), and revealed that the resonances observed under native conditions (i.e., 0 M urea) correspond to the disordered residues: D1-Q18, present in a loop region adjacent to the A-helix, as well as residues attributed to the F-helix and several strands of the A-sheet (Fig. 4a, c, d). Additional resonances could only be resolved upon the addition of urea, with the majority of these resonances (~70%) requiring at least 4 M urea before they became observable (Fig. 4c, d and Supplementary Fig. 3b).

The strong resistance to denaturant indicates that the majority of the AAT-191 polypeptide appears to be compacted with some local regions of structure. These findings are supported by far-UV CD, which shows a propensity for α-helical secondary structure formation of ~26% (Supplementary Fig. S3d, e), and theoretical observations which predict very few regions of hydrophobicity (Fig. 4c). These CD and NMR analyses show that AAT-191 possesses significant secondary structure but lacks persistent tertiary structure. Moreover, the line broadening observed by NMR is also an indication of conformational exchange on a μs-to-ms timescale, and together these data suggest that the fragment adopts a molten globule structure^31^. Consequently, as shown by the similarity of the RNC proteolysis data (Fig. 3a), it is anticipated that the full-length AAT RNCs similarly form a molten globule structure, with compacted N-terminus formed between residues 1–191.

### Single cysteines residues as site-specific reporters of structure formation as probed by PEGylation

**Fig. 5:**
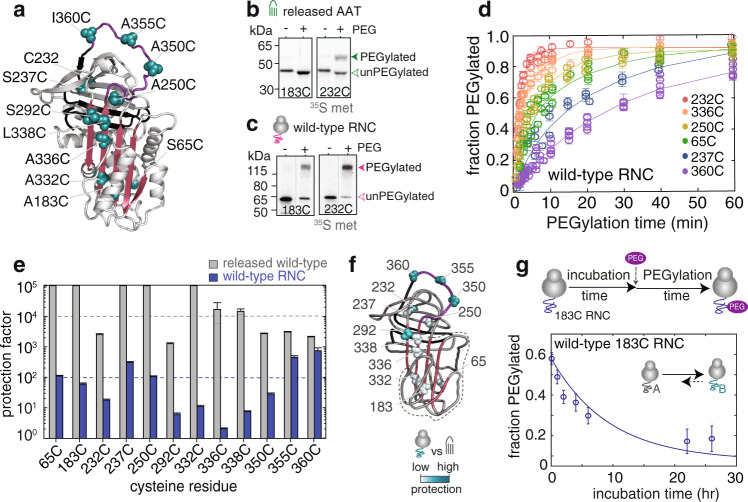
Full-length, ribosome-bound AAT nascent chains form a co-translational folding intermediate. **a** Structure of AAT (1QLP) with all single cysteine probe positions highlighted. **b** Extent of PEGylation in cysteine variants 183C and 232C in released, natively-folded wild-type AAT measured after a 60 min PEGylation reaction at 25 °C. See also Supplementary Fig. 4). **c** As described for **b**, but shows wild-type ribosome-nascent chain complexes (RNC). Source data are provided as a Source Data file. **d** Fitted PEGylation kinetics of selected wild-type RNCs. For clarity, only the first 60 min (of 180 min) are shown (*n* = 4). See Supplementary Fig. 4. **e** Comparison of protection factors of released wild-type AAT and wild-type RNCs at 25 °C. Shown in the dashed lines are the average protection factor values for released wild-type and wild-type RNCs (*n* = 5 biological repeats). **f** Structural depiction of the co-translational folding intermediate of wild-type, derived from a comparison of protections factors against released, natively-folded wild-type AAT. Highlighted in dashed lines is the N-terminal fragment, 1–191 (analysis taken from **e**). **g** (upper) Schematic of a PEGylation reaction for wild-type AAT RNCs highlighting an (RNC) incubation time prior to PEGylation. (lower) Extent of PEGylation for wild-type 183C RNCs as measured across different RNC incubation times. Each data point is a different RNC incubation time-point. (inset) a kinetic model for RNC behaviour as observed over time. (*n* = 5 biological repeats). Data are presented as mean values +/− SEM.

Following the observation that AAT RNCs form persistent structure in the polypeptide segment (1–360) that has emerged from the ribosomal exit tunnel, we examined the structural and dynamic properties of wild-type and Z RNCs more closely. Since rabbit reticulocyte lysate reactions are not readily amenable for producing RNCs in sufficient quantities for NMR structural analysis^32^, we developed PEGylation, a cysteine-mass tagging approach, as an alternative, non-invasive read-out of solvent accessibility in protein structure^33^. Twelve single-cysteine variants of released AAT and corresponding RNCs were engineered, with each cysteine acting as a unique site-specific structural reporter, as defined by their extent of PEG accessibility (Fig. 5a). The cysteine positions were selected on the basis that they were minimally-perturbing to AAT’s stability^34–38^. These also included sites that are known to be solvent-inaccessible in AAT’s natively-folded structure and which could thus serve as folding probes. (Fig. 5b and Supplementary Fig. 4a).

To assess PEGylation as a reliable means of assessing RNC structure formation, we initially examined the structural properties of released, natively-folded AAT, using a PEG moiety (10,000) which cannot enter the ribosomal exit tunnel^33^. We assessed the change of PEG accessibility of each cysteine variant over time at 25 °C and we found that many cysteine sites were largely PEG-inaccessible, but became accessible when the proteins were denatured in urea (Supplementary Fig. 4a). The extent of PEGylation measured after 60 minutes was used to derive protection factor values, as a quantitative measure of solvent accessibility (Fig. 5b, e). These protection factor values were calculated using the model disordered protein, FLN5 (Y719E)^39^, as a reference for a completely unfolded (and thus PEG-accessible) polypeptide under native conditions (see “Methods” section).

Several cysteines (65C, 183C, 237C, 250C, 332C, 336C, 338C) revealed protection factor values of >10,000, and were thus considered PEG-inaccessible within released AAT’s natively-folded structure (Fig. 5e). Other cysteines (232C, 292C, 350C, 355C, 360C) were comparatively more PEG-accessible, with protection factor values ranging between 1000-10,000 (Fig. 5e). Together, these PEGylation results are also consistent with prior characterisation studies of these released AAT cysteine variants^35,36,40–42^, and thus enables us to correlate the extent of protection as measured by PEG-accessibility with the extent of structure formation.

### Full-length AAT forms a co-translational folding intermediate on the ribosome with a propensity to misfold

We then adapted the PEGylation approach to wild-type single cysteine RNC variants which were produced in rabbit reticulocyte lysate and purified with a sucrose cushion. The extent of PEGylation was measured over time (Fig. 5c, d and Supplementary Fig. 4b, c). The wild-type RNCs had protection factor values of ~100 (Fig. 5e). These values contrast those of natively-folded released AAT, which is substantially more protected, and also that of the unfolded model system, which is substantially less protected. This indicates that wild-type RNCs likely form partially-folded structure (Fig. 5e, f). The protection factors also revealed that the wild-type RNC had a comparatively more open N-terminus (65C, 183C) relative to natively-folded AAT (Fig. 5e, f), which is consistent with the molten-globule characteristics observed by NMR for the released N-terminal fragment (AAT 1–191) (Fig. 4a–d). In addition, the wild-type RNC had a more protected C-terminus (292C, 350C, 355C, 360C) relative to released AAT, which is likely related to the proximity that these residues have to the ribosomal tunnel.

The RNC PEGylation profiles also unexpectedly revealed that cysteine modification occurred along a biphasic trend, and where these ribosome-bound nascent chains became less-solvent accessible over time (Fig. 5d and Supplementary Fig. 5a, b). To understand the basis of these phenomena further, we incubated wild-type 183C RNCs at 25 °C, across various times prior to the PEGylation reaction (Fig. 5g and Supplementary Fig. 5a, b). We found that the RNCs were remarkably stable following a 22 h incubation (65% intact); however, the PEGylation extent of the intact RNCs decreased with increasing incubation time (Fig. 5g). Similar results were also obtained when the same analysis was applied to Z 183C RNCs (Supplementary Figs. S4d and S5b). These observations are consistent with a model in which the RNC transitions from one structure (“state A”) towards an alternative PEGylation-resistant structure (<20% PEGylation after a 22 hour incubation, “state B”) with a rate of 0.11 ± 0.02 h^−1^ (Fig. 5g and Supplementary Fig. 5a). The magnitude and rate of this reverse reaction (0.01 ± 0.01 h^−1^) indicates that if the reaction is not reversible, then it at least strongly favours the formation of state B over time. The slow rate of state B’s formation over time alongside the presence of high molecular weight species in gels (Supplementary Fig. 5c), suggests that this process can be reasonably accounted for by a higher order assembly process (released AAT has a polymerisation rate of 0.03 h^−1^^37^). This suggests that wild-type RNCs form a co-translational folding intermediate (state A) with a misfolding (or aggregation) potential (state B).

### Co-translational folding intermediates formed in full-length AAT RNCs persist post-translationally

**Fig. 6:**
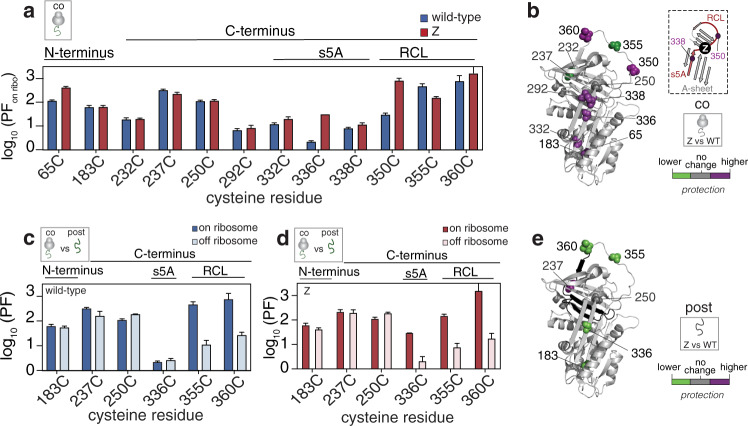
Co-translational folding intermediates persist post-translationally. **a** Protection factors (PF) calculated for the co-translational folding intermediates formed by wild-type and Z RNCs as derived from PEGylation kinetics (*n* = 5 biological repeats). **b** Difference in protection in the Z’s co-translational folding intermediate relative to that of wild-type, as mapped onto AAT’s native structure. (inset) A magnified view of AAT’s central A-sheet region, highlighting the location of the s5A/RCL region and the Z mutation. This region is implicated in AAT folding (see text). **c** A comparison of the protection factors for wild-type’s co-translational folding intermediate (on ribosome) and post-translational folding intermediate (off ribosome) for a series of cysteine positions. **d** As described for **c** but shows Z. **e** Difference in protection in Z’s post-translational intermediate relative to that of wild-type, as mapped onto AAT’s native structure. Data are presented as mean values +/− SEM.

To analyse the structural characteristics of the co-translational folding intermediate identified above by PEGylation in ribosome-bound Z nascent chains, we compared the state A protection values to those of wild-type RNCs (Fig. 6a and Supplementary Fig. 4c, d) (see Supplementary Figs. 6 and 7 for information on state B’s characteristics). We found that the values for Z were, on average, ~28% higher relative to those of wild-type, suggesting that they have regions of increased protection (Supplementary Fig. 7a). A closer inspection of the individual cysteines revealed very modest, but nevertheless discernible local differences in protection (Fig. 6a): the N-terminal region (65C, 183C) is somewhat unperturbed, whilst probes in close proximity (336C, 338C) to the Z mutation site (E342K) show more protection. Generally, Z RNC’s C-terminus, including 292C and the region spanning 332C to 360C, is also more protected (Fig. 6a,b) except for 355C which is marginally less protected. This latter region corresponds to strand 5A of the A-sheet and the reactive centre loop (“s5A/RCL”)^43,44^, and is known to be essential for the closure of the A-sheet during folding to avoid misfolding^40^; the s5A/RCL segment is also the site of the Z mutation (Fig. 6a, b).

We next explored whether the observed structural characteristics of the co-translational folding intermediate could be observed post-translationally. To recapitulate a post-translational folding intermediate, we applied the PEGylation approach to released AAT that had been unfolded in 3 M urea, as an analogue. 3 M urea was selected because it is a condition at which AAT’s folding intermediate is substantially populated at equilibrium^45^. As was previously observed for the RNCs, the PEGylation kinetics were similarly biphasic in nature (Supplementary Fig. 8b–e), forming a more protected state over time, which is consistent with polymerisation (Supplementary Fig. 8f and see ref. ^46^). On this basis, we calculated protection factor values for the post-translational folding intermediates of both wild-type and Z AAT using a kinetic model similar to that derived for the RNCs as described above (see “Methods” section).

An analysis of the protection factor values observed for wild-type’s post-translational folding intermediates show marginal differences compared to the co-translational folding intermediate at the N and C-termini: the N-terminus (183C) remains relatively unchanged and the C-terminus (237C, 355C, 360C), was found to be generally less protected in the post-translational intermediate (Fig. 6c). This suggests that the ribosome-occluded segment, I360-K394, has become solvent exposed following nascent chain release, but is not, however, integrated into the structure as a C-terminal hairpin since the reporter of this region, 250C, shows small differences in the co- and post-translational intermediates (Fig. 6c and Supplementary Fig. 8d), but is highly-protected in the natively-folded protein (Fig. 5e). Z’s post-translational intermediate shows a similar trend to that observed for wild-type although unlike wild-type, Z’s post-translational intermediate generally shows less protection in both its N-termini and C-termini compared to its co-translational intermediate (Fig. 6c, d and Supplementary Fig. 8d). There are also differences observed in reporters of the s5A/RCL region, particularly in 336C (a reporter of the Z mutation site) and 360C, which show that this segment is comparatively less protected in Z’s post-translational intermediate relative to that of wild-type (Fig. 6d, e).

Overall, these results suggest that immediately following release from the ribosome, a large extent of the nascent chain structure that forms co-translationally persists post-translationally. Additionally, the characteristics observed for Z suggest that it forms a distinct co-translational folding intermediate, with small but discernible differences in its structure and/or dynamics relative to wild-type.

### Co-translational folding governs post-translational folding outcomes

**Fig. 7:**
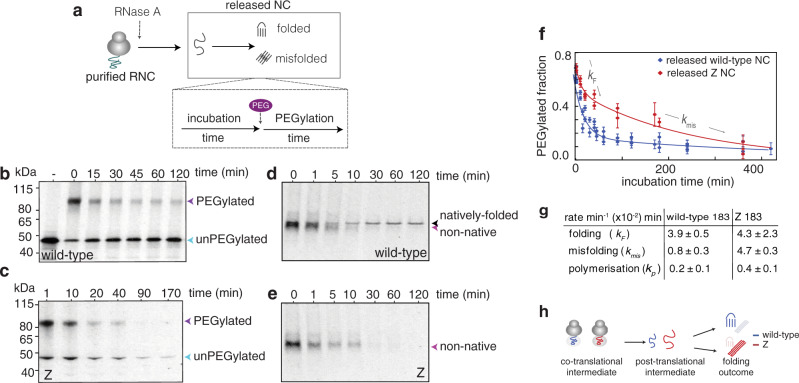
Monitoring post-translational folding outcomes of released AAT nascent chains (NC). **a** A reaction scheme for monitoring post-translational folding by PEGylation, in which nascent chains are synchronously-released from 183C RNCs using RNase A to initiate their (off the ribosome) folding. PEG is added at intervals to monitor the folding process, and the PEGylation reaction time is 60 min at 25 °C. (*n* = 5 biological repeats). **b** PEGylation of RNase A-released nascent chains of wild-type 183C monitored via partially-denaturing PAGE. **c** As described for **b**, but shows Z 183C. **d** Folding of RNase A-released, wild-type nascent chains as monitored by native PAGE (equivalent samples as shown in (b), but without PEGylation). **e** Folding of RNaseA-released, Z nascent chains as monitored by native PAGE (equivalent samples as shown in **c**, but without PEGylation). **f** A PEGylation profile for RNAse A-released wild-type and Z 183C nascent chains from their respective RNCs (analysis of **b**, **c**, respectively). Data are presented as mean values +/− SEM. **g** Rates of post-translational folding, misfolding and polymerisation of RNase A-released 183C nascent chains (*n* = 4 biological repeats). **h** A schematic illustrating the tempering effect imposed by ribosome on emerging nascent chains which is removed after the nascent chains are released. All PAGE used ^35^S methionine detection.

Finally, we studied whether co-translational folding on the ribosome directly influences post-translational folding outcomes for the released AAT nascent chains. Starting from purified, full-length AAT RNCs in buffer to decouple folding processes from translation, we measured the post-translational folding of synchronously-released nascent chains that arise from a homogenous AAT population, by treating the RNCs with RNase A. We combined PEGylation (Fig. 7a–c) and native PAGE analysis (Fig. 7d, e) on 183C as the site-specific reporter of folding, owing to it being accessible in the (co/post) folding intermediate (Fig. 6c) and is buried in AAT’s native structure (Fig. 5e and see ref. ^36^). The PEGylation kinetics for the synchronously-released, wild-type (and Z) nascent chains revealed two rates (Fig. 7f, g): *k*_F_ is a fast rate (minutes) consistent in magnitude to an intermediate-to-native folding transition observed previously in released AAT using tryptophan fluorescence^47^. This phenomenon is corroborated by the ~70% PEGylation (Fig. 7f) extent in wild-type nascent chains, which is comparable to that of the RNC (~60%, (Fig. 5g)), and thus suggests that the newly-released nascent chains possess a similarly partially-folded structure upon immediate release. Additionally, the decrease in PEGylation signal coincides with the appearance of natively-folded species (Fig. 7b, d). The observed second rate (*k*_mis_), is slower (hours), and is consistent with a misfolding process that facilitates polymerisation (*k*_P_) (Fig. 7f, g). We also observe that immediately following release (1 min) the majority of the wild-type nascent chains migrated as non-native species for up to 5 min, prior to completing their native fold, as monitored by native PAGE (Fig. 7d); for Z however, the non-native species appeared to persist for a longer time period in this conformation (>10 min) and a natively-folded species was not observed within this 60 min time frame (Fig. 7e and see ref. ^47^).

Interestingly, both wild-type and Z show similar rates of folding, but the misfolding rate of Z is enhanced compared to wild-type (Fig. 7g). This result is reflected in the extent of native folding success, which was 82.9 ± 0.1% for wild-type (derived from PEGylation kinetic rates (Fig. 7d)). By contrast, Z’s apparent folded population (47.2 ± 34.1%) likely reflects a kinetically-trapped, non-native species, rather than a natively-folded structure (Fig. 7e). The substantial differences in post-translational folding outcomes of newly-released wild-type compared to Z suggests that during nascent chain emergence, the ribosome likely sequesters the altered properties of Z’s co-translational folding intermediate; following nascent chain release, however, this “hold” is relieved and Z’s altered folding intermediate readily promotes post-translational misfolding (Fig. 7h).

## Discussion

Our systematic investigation of AAT biosynthesis has revealed its capacity to form persistent full-length RNCs which are capable of forming a co-translational compact structure, and which influences folding outcomes for a nascent chain upon its release. Although the molecular basis for AAT stalling remains unclear, it is likely that such arrest is an inherent property of this system that may allow for more efficient co-translational glycosylation. It is anticipated that such arrest may extend to other proteins, where in conjunction with slow rates of translation, may be a means of permitting co-translational events such as folding, assembly, modifications, or transport to take place. Limited proteolysis, PEGylation and NMR spectroscopy each revealed that these RNCs can form a co-translational folding intermediate with a compacted N-terminus and molten-globule properties in residues 1–360, that have emerged from the ribosomal exit tunnel. We also found from PEGylation analyses that these RNCs also have an intrinsic propensity to engage in higher-order association (state B), anticipated to be driven by the ribosome.

We also evaluated AAT’s pathological variant, Z, to explore why it is more prone to misfolding. A comparative analysis using PEGylation showed only very modest differences between wild-type and Z’s co-translational folding intermediates. This is indicative of the ribosome likely being able to temper the dynamic properties of the tethered nascent chain, probably through imposing steric effects^48^ via surface interactions^39,49^, and within the cell, likely includes interactions with auxiliary proteins e.g., molecular chaperones^1,50^. This tempering mechanism is potentially one means of mitigating the possible deleterious effects of misfolding during biosynthesis, particularly for cytosolic proteins, while for AAT, it is anticipated that ribosome tethering and surface interactions within the endoplasmic reticulum environment may prevail. This imposition by the ribosome is, however, relieved upon nascent chain release; co-translationally-formed structure persists post-translationally causing wild-type and Z newly-released nascent chains to follow divergent folding outcomes. This divergence is observed in the substantial differences in post-translational folding kinetics (and outcomes), since wild-type and Z AAT share near-equivalent native-state stabilities^14,36^. Z AAT’s inability to fold in a timely manner off the ribosome is governed by the altered kinetic properties of its post-translational folding intermediate immediately following ribosome release. This finding is supported in part, by studies of small molecule “folding correctors” of (released) AAT which bind to, and alter Z AAT during its folding^16,51^. Additionally, evidence from RNC PEGylation shows that perturbations exist in both Z’s co-translational and post-translational intermediates which suggest that like folding, AAT’s misfolding propensity also begins co-translationally; the origin of Z’s “kinetic folding defect” thus first develops on the ribosome.

**Fig. 8:**
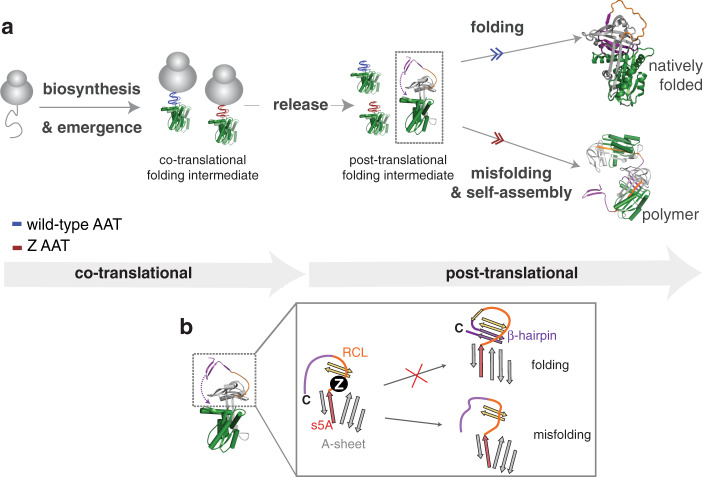
Co-translational and post-translational folding and misfolding AAT. **a** A folding and misfolding model for AAT during biosynthesis. Highlighted in green is the 1–191 fragment that forms co-translational structure on the ribosome. **b** A schematic showing how Z’s kinetic folding defect, which begins developing co-translationally, guides post-translational folding outcomes. Shown is a magnified view of the A-sheet/RCL region in the post-translational intermediate: A-sheet (grey, and strand 5A (s5A) in red), B-sheet (gold), reactive centre loop (RCL), orange, β-hairpin (purple). The insertion of the final 35 C-terminal residues to form a β-hairpin in AAT’s core and complete the native fold, is a key component of the folding/misfolding partition.

From our analyses, we propose a folding model in the endoplasmic reticulum (Fig. 8a): AAT begins folding co-translationally on the ribosome and whereupon nascent chain release, the compacted N-terminal region provides a sufficient scaffold to promote subsequent native structure formation. This latter process includes formation of the C-terminal B-sheet and C-sheet and closure of the central A-sheet. These steps are likely strongly influenced by the formation of s5A, and the threading of 35 C-terminal amino acids that were previously ribosome-occluded, to form a C-terminal β-hairpin (K365–K394) into the core of the protein to complete the B-sheet, which is nestled behind the A-sheet. This hypothesis is consistent with our PEGylation observations of a less protected C-terminus in the post-translational folding intermediate, and is supported by hydrogen-deuterium exchange studies of released AAT^15^. The Z mutation introduces small, but discernible changes in the co-translational folding intermediate which persists post-translationally to modulate the kinetics of the intermediate-to-natively-folded state transition. This modulation likely relates to the delayed formation of the C-terminal β-hairpin, which is adjacent to the destabilised s5A/RCL region and Z mutation site (Fig. 8b). As a consequence, Z nascent chains persist post-translationally in a trapped, partially-folded intermediate (as observed by native PAGE) which is vulnerable to self-assembly. On account of its structural properties, self-assembly likely proceeds via a C-terminal-based AAT polymerisation mechanism as observed in hepatocytes^12^ (Fig. 8 and Supplementary Fig. 9). AAT is an archetypal serine proteinase inhibitor, and other serpins (antichymotrypsin^52^, plasminogen activator inhibitor 1^53^, and antithrombin^54,55^) are similarly known to fold via misfolding-folding prone intermediates and polymerise. It is highly likely therefore, that the folding phenomena described for AAT can be broadly extended across the serpin superfamily, but where the specific details will differ according to amino acid sequence.

Similarly, it is anticipated that the observed relationship between co-translational and post-translational folding shown here for AAT, may also be shared by proteins which complete their native folds under kinetic control via the formation of protein folding intermediates, or in those proteins with complex three-dimensional topologies^56–59^. In both of these cases, co-translational folding intermediates likely reduce the conformational space required to promote efficient folding. Moreover, the ribosome may be capable of mitigating co-translational misfolding processes as the nascent chain progressively emerges, however as shown for Z AAT, aberrant folding is only temporarily suspended on the ribosome since post-translational misfolding readily occurs; within the cell if not maintained by quality control mechanisms in the endoplasmic reticulum, an abundance of these misfolded, released nascent chains can promote rampant polymerisation and aggregation. It is also envisaged that the observation of co-translational misfolding in Z AAT may also be representative of a general mechanism that promotes domain-swapping during early biosynthesis.

Broadly, this study also contributes to our understanding of how the ribosome is emerging as a key player in the aetiology of conformational diseases such as alpha-1-antitrypsin deficiency, cystic fibrosis^56^, and Huntington’s disease^60^. As shown here, elucidating both the structural characteristics of co-translational folding intermediates and determining their impact on promoting post-translational structure formation may be a basis for designing targeted strategies that mitigate nascent chain misfolding as it first begins on the ribosome itself.

## Methods

### Generation of AAT DNA constructs for ribosome-nascent chain complexes

Sequence corresponding to the residues D1 to K394 of mature wild-type and Z AAT was sub-cloned into the pLDC vector^61^ (for RNCs), with single cysteine variants generated on a C232S background^37^. Linearised DNA templates used for the in vitro synthesis of released AAT and AAT RNCs were amplified using a T7 forward oligonucleotide and a reverse oligonucleotide specific to the AAT sequence. Please refer to Supplementary Table 1 for all the oligonucleotide sequences. Following PCR, the samples were treated with DpnI and purified further. The purity of the linearised DNA was assessed by agarose gel electrophoresis.

### Generation of released protein and RNCs using a coupled rabbit reticulocyte lysate system (RRL)

AAT was synthesised in vitro using TNT T7 Quick Coupled Transcription/Translation system (Promega) following the manufacturer’s instructions. The reactions were initiated by the addition of linearised DNA templates and ^35^S methionine, and were quenched with either 1 mg/mL RNase A, or 1 mg/mL cycloheximide; used to preserve any tRNA-bound species. Released AAT produced in the cell-free reactions was partially purified via Ni-NTA spin columns according to the manufacturer’s protocol. AAT RNCs were produced using DNA constructs that lacked a stop codon. AAT RNCs and 80S ribosomes were purified via sucrose cushion ultracentrifugation using a 30% (w/v) sucrose cushion prepared in RNC buffer (20 mM HEPES/KOH, pH 7.5, 100 mM potassium acetate, 14 mM magnesium acetate), spun at 350,000×*g* using a TLA-120.2 rotor (Beckman Coulter) for 60 min at 4 °C. RNC pellets were gently washed once with PEGylation buffer (20 mM HEPES/NaOH, pH 7.2, 100 mM NaCl, 5 mM MgCl_2_) before resuspension in PEGylation buffer (unless indicated otherwise). Visualisation of proteins incorporated with ^35^S methionine was performed using autoradiography^62^. For endoplasmic reticulum targeting experiments, released AAT with its native signal sequence (MPSSVSWGILLLAGLCCLVPVSLAE) was expressed in nuclease-treated rabbit reticulocyte lysate in the presence of canine pancreatic microsomes (Promega) following the manufacturer’s instructions. After microsome purification^23^, the purified N-glycosylated species were deglycosylated under denaturing conditions (100 °C) with the glycoaminidase, PNGase F (New England Biolabs), to remove the N-glycans.

### Production of released AAT from *E. coli*

Uniformly ^1^H,^15^N- labelled released wild-type AAT was produced using the pQE31 vector in XL2Blue *E.coli*, and purified using Ni-metal affinity, anion-exchange (Q sepharose), and size exclusion (Superdex 200 16/600) chromatographic steps^63^. For the production of AAT-191, the protein was expressed using the pLDC vector^61^ in LOBSTR *E.coli* strain (Kerafast) and purified using Ni-IDA metal affinity chromatography and size exclusion (Superdex 75 16/600) chromatography.

### Analysis of RNCs and released nascent chains using PAGE

For visualising the tRNA-bound nascent chain species in RNCs and PEGylated RNC samples, low pH, partially-denaturing conditions were used^32,64^. Samples were mixed with 2× SDS-containing loading dye (125 mM Bis–Tris, 0.04% (w/v) bromophenol blue, 4% (w/v) SDS, 20% (w/v) glycerol, pH 5.7) and were not boiled. The samples electrophoresed using 8% (w/v) acrylamide/bis-acrylamide gels and MOPS running buffer (2.5 mM Tris-base, 2.5 mM MOPS, 0.005% (w/v) SDS, pH 7.7). DTT was added fresh prior to sample loading. Denaturing PAGE (i.e., Laemmli SDS-PAGE) was used to visualise released nascent chains, released proteins, and limited proteolysis experiments, where samples were mixed with SDS dye and boiled before loading onto Bis-Tris based gels. For native PAGE conditions, samples were mixed with 2× native loading dye (124 mM Tris-Cl, 20% (w/v) glycerol, 0.02% (w/v) bromophenol blue, pH 6.8) and were electrophoresed using 10% (w/v) acrylamide/bis-acrylamide gels, on ice, and using a discontinuous buffer system comprised of an anode (100 mM Tris-Cl, pH 7.8) and cathode buffer (53 mM Tris-base, 68 mM glycine, pH 8.9).

### Monitoring the kinetics of synthesis and folding during biosynthesis of released AAT in rabbit reticulocyte lysate (RRL)

Transcription was initiated by addition of the linear DNA template of full-length, released AAT to the RRL (lacking ^35^S-methionine). After a 10 min incubation at 30 °C, ^35^S-methionine was added to initiate translation and after 45 s the initiation inhibitor, aurintricarboxylic acid, was added to a final concentration of 75 µM to prevent the initiation of further translation events. During a 60 min incubation at 30 °C, aliquots were withdrawn from the reaction at different times and quenched with RNase A. Samples were subsequently analysed by partially-denaturing and native PAGE and autoradiography. For the analysis of biosynthesis and folding, 60 min was chosen as a relative end-point to monitor the accumulation of proteins over time.

### Activity of released AAT nascent chains

Wild-type AAT RNCs were purified through a 30% (w/v) sucrose cushion, treated with 0.5 mg/mL RNase A and incubated at 25 °C for 4 h to allow released nascent chains to fold to their native structure. Samples were then treated with increasing concentrations of chymotrypsin (0–250 nM) in chymotrypsin buffer (20 mM HEPES/NaOH, pH 7.2, 100 mM NaCl, 5 mM MgCl_2_, 10 mM CaCl_2_) for 5 min at 25 °C, before being quenched with 1 mM PMSF and analysed by denaturing PAGE.

### Measurement of the biosynthesis rate of released AAT in rabbit reticulocyte lysate

The accumulation of released protein over synthesis time was quantified by densitometric analysis, and fitted to Eq. 1.

1 $$\frac{P[t]}{{P}_{{{\max }}}}=\left\{\begin{array}{cc}0 & {{{\rm{if}}}}\,t\le {t}_{0}\\ 1-{e}^{-r(t-{t}_{0})} & {{{\rm{if}}}}\,t > {t}_{0}\end{array}\right.,$$

where *P[t]/P*_max_ is the fraction of synthesised protein at time *t* relative to the total product *P*_max_ made at 60 mi; *t*_0_ is the lag time between the addition of methionine (i.e., translation initiation) and when the first full-length product was synthesised; *r* is the average rate of protein accumulation. To calculate the rate of synthesis, the protein length (i.e., 394 amino acids) was divided by the time it takes to completely synthesise one protein molecule (i.e., 1/*r*).

### Limited proteolysis using proteinase K

Following wild-type and Z RNCs synthesis, the reactions were quenched with 1 mg/mL of cycloheximide on ice, and proteinase K (solubilised in PEGylation buffer) was added rapidly at a concentration of 4 ng/μL. Aliquots were taken at different time points and quenched with 1 mM PMSF prior to the samples being analysed by partially-denaturing PAGE. For the analysis of intact RNCs, RNC samples following biosynthesis were purified through a 30% (w/v) sucrose cushion and resuspended in PEGylation buffer, to which proteinase K was added to an effective concentration of 0.2 ng/µL. Reactions were incubated on ice, aliquots were withdrawn at different time points and immediately quenched with 1 mM PMSF. Samples were subsequently analysed by partially-denaturing PAGE and autoradiography. To identify proteinase K-digested fragments with intact N-termini, AAT RNCs purified from rabbit reticulocyte lysate and released AAT (1 µM) purified from *E. coli* were subjected to an effective concentration of 1 ng/µL proteinase K in PEGylation buffer. After 1–3 min of incubation on ice for AAT RNCs, and 2–6 min for released AAT, proteolysis was quenched with the addition of PMSF to an effective concentration of 10 mM. Samples were then analysed by PAGE followed by an anti-His western blot.

### Evaluating the stability of AAT RNCs using limited proteolysis

The proportion of tRNA-bound RNC over time, relative to total amount at the start of the limited proteolysis reaction, was quantified by densitometric analysis and fitted to Eq. 2.

2 $$\frac{I[t]}{{I}_{{\max }}}={e}^{-{k}_{{{{{\rm{LP}}}}}}t}$$

where *I[t]/I*_max_ is the proportion of intact AAT RNCs at time *t* relative to the total amount *I*_max_ at the start of reaction; *k*_LP_ is the rate of degradation.

### PEGylation of purified ribosome-nascent chain complexes

Wild-type and Z RNC cysteine mutants were subjected to PEGylation where the extent of PEGylation was monitored as a function of PEGylation time. Following a 60 min biosynthesis reaction in rabbit reticulocyte lysate, the RNCs were purified through a 30% (w/v) sucrose cushion, resuspended in PEGylation buffer, immediately after which the samples were treated with an equal volume of 2 mM mPEG-maleimide (PEG, mPEG-mal) 10,000 (final concentration 1 mM). For some RNCs whose rates of PEGylation were too fast (292C, 338C), PEGylation was performed with 0.1 mM mPEG-mal (final concentration). The RNCs were incubated at 25 °C during which aliquots were withdrawn from the reaction at different PEGylation times and the PEGylation reaction was quenched with 100 mM DTT. Samples were subsequently subjected to partially denaturing PAGE and visualised using autoradiography. For PEGylation experiments with an RNC incubation time, wild-type and Z 183C RNCs were purified through a sucrose cushion and resuspended in PEGylation buffer as described above. Samples were then incubated at 25 °C for a time between 0 and 26 h before the addition of 1 mM mPEG-mal. Aliquots were again withdrawn at different PEGylation times from the reaction and quenched with 100 mM DTT. To avoid the variation of PEG quality affecting the analysis, wild-type and Z experiments were conducted in parallel on the same day. To compare the solvent-accessibility of released wild-type AAT relative to the RNCs, PEGylation kinetics were converted into protection factors, a measurement used in hydrogen-deuterium exchange studies^65,66^. Here, since PEGylation of a cysteine is highly dependent on a range of factors^33^, an intrinsic PEGylation rate was not possible to measure. Instead, PEGylation of the natively-unfolded model protein, FLN5 Y719E was used as a reference, since its single cysteine, C747, is completely solvent exposed and undergoes rapid PEGylation (90% PEGylation within 5 s). The PEGylation of this cysteine is described by Eq. 3.

3 $$-\frac{1}{t}{{{\rm{log }}}}(1-{{{{\rm{FractionPegylated}}}}}).$$

### PEGylation of the post-translational intermediate

Single cysteine AAT RNC variants were purified by sucrose cushion as described above. The nascent chains were released with 1 mg/mL RNase A and immediately unfolded in a final concentration of 6 M urea a concentration sufficient to unfolded AAT^45^. An equal volume of 2 mM mPEG-mal 5000 was added to the reaction (final concentration 1 mM) to initiate refolding in a final concentration of 3 M urea. For cysteine variants for which the PEGylation rates were too fast (336C, 355C, 360C) to measure with 1 mM mPEG-mal, a final concentration of 0.5 mM was used. Analysis of the PEGylation kinetics was performed as described for the RNCs, aside from 250C which was fitted to a single exponential.

### Monitoring post-translational folding of synchronously-released nascent chains via native PAGE and PEGylation

183C RNCs were produced in rabbit reticulocyte lysate and subsequently purified from the lysate using a 30% (w/v) sucrose cushion as described above. The pellet was resuspended in PEGylation buffer supplemented with 50 µM DTT to fully reduce all cysteines, and 0.5–1 mg/mL RNase A to synchronously release all nascent chains. Samples were then incubated for various RNC incubation times at 25 °C after which half of each sample was snap-frozen in liquid nitrogen and folding was assessed by partially-denaturing PAGE. The remaining half of the samples were subjected to 1 mM mPEG-mal (dissolved in PEGylation buffer just prior to use) for 1 h at 25 °C. The PEGylation reaction was quenched with 100 mM DTT, and the samples analysed by partially-denaturing PAGE.

### Quantification of folding rate of released proteins in lysate, and released nascent chains in buffer

Native PAGE was used to monitor the rate of folding of released proteins in lysate during biosynthesis, and nascent chains released (in buffer) from RNCs using RNase A treatment. To “quench” folding, aliquots were immediately snap-frozen with liquid nitrogen, and the samples were quickly thawed on ice immediately prior to loading onto a native PAGE (in reverse order). Fractions of natively-folded species at time *t*, relative to the maximum amount observed at 60 min, was quantified by densitometric analysis, and fitted to Eq. 1. In this experiment, *t*_0_ is the lag time between the RNase A treatment (initiation of post-translational folding) and when the first natively folded species was observed; *r* is the average rate of accumulation of native species, here defined as the folding rate.

### Western blot analysis of AAT RNCs and released proteins

Anti-His western blots of AAT RNCs and released proteins were performed using the penta-His antibody (1:5000 dilution) (Qiagen, catalogue number 34460), following the manufacturer’s instructions. For the detection of AAT-191, the anti-antitrypsin mouse monoclonal antibody 1C2 (1:5000 dilution) (Sigma, catalogue number SAB4200198) was also used. Western blots were imaged using Image Studio (v 4.0)

### Densitometric analysis of polyacrylamide gels

For densitometric analysis of polyacrylamide gels and autoradiographs, ImageJ^67^ and Fityk^68^ were used to quantify the intensity of protein bands separated on either partially-denaturing or native PAGE gels. Densitometry was carried out on exposures within the linear dynamic range of the film. Errors in densitometric analysis were derived from Fityk.

### Fitting and analysing PEGylation data for the cysteine RNC mutants

The following reaction scheme was used to describe a scenario for the PEGylation of an RNC following an incubation time period (*t*_RNC_) (Supplementary Fig. 10a). Freshly purified RNCs have an RNC incubation time (*t*_RNC_) of zero. The PEGylation data of wild-type and Z RNC were fitted to a numerical solution of differential equations describing a reversible transition of state A to B (with rates *k*_AB_ and *k*_BA_) within the RNC co-translational intermediate ensemble, following a 60 min biosynthesis reaction. *A*_0_ and *B*_0_ are relative proportions of states A and B at *t*_RNC_ = 0 (i.e., immediately after synthesis); *k*_PA_ and *k*_PB_ are distinct PEGylation rates for states A and B at a specific cysteine position. All parameters are fitted globally for all 12 cysteine mutants, but the wild-type and Z datasets were fitted separately to avoid making any assumptions about the relationship between wild-type and Z RNCs. A fraction of any inactive PEG was also incorporated into the numerical solution, as a constant, alpha.

### Fitting and analysis of PEGylation data for the post-translational intermediate

A similar reaction scheme as described for the RNC was used to rationalise and describe the biphasic trend seen for most cysteine probes in the urea-induced post-translational intermediate studied (Supplementary Fig. 10b). Here, t_NC_ is the length of time released, unfolded nascent chains are incubated in 3 M urea prior to a 1 h PEGylation reaction (*t*_p_ = 60 min). Experiments measuring the extent of PEGylation over time are described when t_NC_ = 0. The PEGylation data of the wild-type and Z post-translational intermediates were fitted to a numerical solution of differential equations describing a transition of state A to state B (with rate k_B_), which then undergoes a slow transition towards a polymerised state, X (with rate *k*_X_). *k*_PA_ and *k*_PB_ are distinct rates for states A and B at each cysteine position. The intermediate state (state A) was chosen as the initial state in the reaction scheme as it was assumed to be almost instantaneously populated from the unfolded state due to the fast rate of refolding from 6 to 3 M. In the initial state, the intermediate was assumed to be populated entirely by state A which converts to B over time.

### CD spectroscopy

AAT proteins were prepared in 25 mM Na_2_HPO_4_, 50 mM NaCl, pH 7.4 and far-UV scans (190–250 nm) were collected with 0.1 nm increments at 25 °C. Data were collected and analysed using Chirascan software (v. 4.7.0.194)^69,70^.

### NMR spectroscopy

NMR samples of ^15^N-labelled AAT-191 (190 µM) were prepared in 25 mM Na_2_HPO_4_, 50 mM NaCl, pH 7.4 in 10% (v/v) D_2_O and 0.001% (w/v) DSS. NMR data were acquired at 283 and 298 K on 800-MHz and 950-MHz Bruker Avance III HD spectrometers equipped with a TXI cryogenic probe. Two-dimensional ^1^H-^15^N SOFAST-HMQC spectra^71^ were acquired with 256 complex points and sweep widths of 33 ppm in the indirect dimension, and 3072 complex points and sweep widths of 19 ppm in the direct dimension. Spectra were referenced to DSS^72^ and processed with NMRPipe^73,74^. The backbone amide assignment of AAT-191 was acquired in 8 M urea at 298 K using triple resonance experiments (HNCO, HN(CA)CO, HN(CO)CACB and HNCACB) recorded with a 25% sampling schedule. Assignments were then transferred to spectra acquired in progressively reduced concentrations of urea.

### Statistics and reproducibility

All experiments were conducted with at least three biologically independent repeats or as indicated in the text. All data was reproduced with similar results, as described by error bars.

### Reporting summary

Further information on research design is available in the Nature Research Reporting Summary linked to this article.

## Supplementary information

Supplementary Information

Reporting Summary

## Data Availability

All data generated in this study are available within the Article, Supplementary Data and Source Data files. Source data are provided with this paper.

## Source data

Source Data
