# Supplementary material for: Nascent chains can form co-translational folding intermediates that promote post-translational folding outcomes in a disease-causing protein
Source: Nat Commun. 2021 Nov 8;12:6447. doi: 10.1038/s41467-021-26531-1 (PMC8576036; doi:10.1038/s41467-021-26531-1)
Supplement: Supplementary file 1 — Supplementary Information [file 41467_2021_26531_MOESM1_ESM.pdf]

Supplementary Table 1: Shows the oligonucleotide sequences used to generate the single cysteine AAT variants, and also the oligonucleotides used to amplify linear DNA (see Methods).

Name	Sequence (5' to 3')	Notes
T7 promoter forward	CTCGATCCCGCGAAATTAATACG	pET21b(+) T7 sequence
AAT RNC reverse	TTTTTGGGTGGGATTCACCACTTT	full length AAT with no stop codon
isolated AAT reverse	GGCGAGCTCTTATTTTTGGGTGGGATT	full length AAT with stop codon
S65C forward	TTTGCAATGCTCTGCCTGGGGACCAAG	single cysteine
S65C reverse	CTTGGTCCCCAGGCAGAGCATTGCAAA	single cysteine
A183C forward	GACACAGTTTTTTGCCTGGTGAATTAC	single cysteine
A183C reverse	GTAATTCACCAGGCAAAAAACTGTGTC	single cysteine
S237C forward	AAGAAGCTGTCCTGCTGGGTGCTGCTG	single cysteine
S237C reverse	CAGCAGCACCCAGCAGGACAGCTTCTT	single cysteine
A250C forward	GGCAATGCCACCTGCATCTTCTTCCTG	single cysteine
A250C reverse	CAGGAAGAAGATGCAGGTGGCATTGCC	single cysteine
C232S forward	TTTAACATCCAGCACTCTAAGAAGCTGTCCAGC	single cysteine
C232S reverse	GCTGGACAGCTTCTTAGAGTGCTGGATGTTAAA	single cysteine
S292C forward	TTACCCAAACTGTGCATTACTGGAACC	single cysteine
S292C reverse	GGTTCCAGTAATGCACAGTTTGGGTAA	single cysteine
A332C forward	AAGCTCTCCAAGTGCGCCGTGCATAAG	single cysteine
A332C reverse	CTTATGCACGGCGCACTTGGAGAGCTT	single cysteine
A336C forward	GCCGTGCATAAGTGCGTGCTGACCATC	single cysteine
A336C reverse	GATGGTCAGCACGCACTTATGCACGGC	single cysteine
L338C forward	CATAAGGCTGTGTGCACCATCGACGAG	single cysteine
L338C reverse	CTCGTCGATGGTGCACACAGCCTTATG	single cysteine
E342K forward	CTGACCATCGACAAAAAAGGTACTGAA	single cysteine
E342K reverse	TTCAGTACCTTTTTTGTCGATGGTCAG	single cysteine
A350C forward	GAAGCTGCAGGGTGCATGTTTTTAGAG	single cysteine
A350C reverse	CTCTAAAAACATGCACCCTGCAGCTTC	single cysteine
A355C forward	ATGTTTTTAGAGTGCATACCCATGTCT	single cysteine
A355C reverse	AGACATGGGTATGCACTCTAAAAACAT	single cysteine
I360C forward	ATACCCATGTCTTGCATCCCCCCCGAG	single cysteine
I360C reverse	CTCGGGGGGGATGCAAGACATGGGTAT	single cysteine

a

wild-type AAT biosynthesis	
Rate of protein accumulation (min^{-1})	0.18± 0.02
Lag time of biosynthesis (min)	1.83 ± 0.32
Apparent rate of biosynthesis (aa/s)	1.16 ± 0.15
Folding rate in lysate (min^{-1})	0.096 ± 0.012
Lag time of folding in lysate (min)	3.58 ± 0.64
**Z AAT biosynthesis**	
Rate of protein accumulation (min^{-1})	0.51 ± 0.16
Lag time of biosynthesis (min)	1.36 ± 0.39
Apparent rate of biosynthesis (aa/s)	3.32 ± 1.07
Rate of second process (min^{-1})	0.0026 ± 0.0007

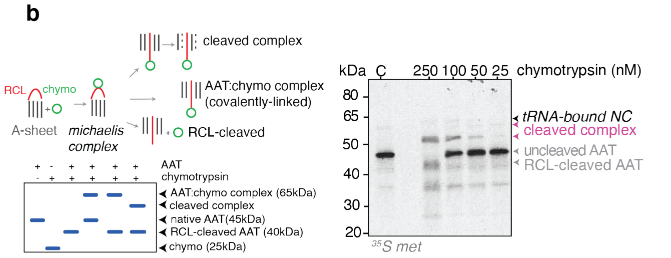

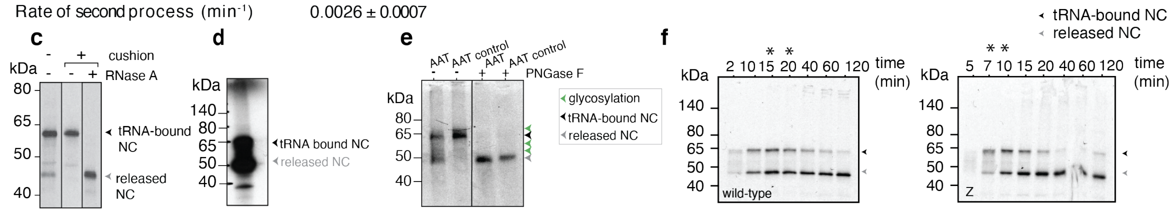

Supplementary Figure 1: Biosynthesis and folding of released AAT in rabbit reticulocyte lysate (RRL). (a) Table showing rates of biosynthesis and folding for released wild-type AAT in RRL and the rates of biosynthesis only for released Z AAT. Rates were normalised to 60 minutes (the length of the biosynthesis reaction) to account for differences in levels of exposure during autoradiography between different films, and possible differences in levels of expression between coupled transcription-translation reactions. Z biosynthesis was fit to a double exponential; the first rate (fast) is an apparent rate of biosynthesis and the second slower rate correlates to a loss of the full-length material, likely due to polymerisation (see Fig. 1). We report here apparent rates of biosynthesis to represent a cumulative rate, rather than absolute translation rates. This is to account for additional processes, for example related to tethering (see f). (b) (left) Schematic showing AAT's interaction with the serine proteinase, chymotrypsin (chymo) and its analysis via denaturing PAGE. (right) Denaturing PAGE with ^{35}S methionine detection of newly-synthesised and released nascent chains were incubated with increasing concentrations of chymotrypsin (chymo), as an orthogonal means of probing structure formation. Under these conditions, a cleaved AAT:chymo complex[62], along with cleaved AAT is detected. This result suggests that the AAT had acquired its canonical, native fold and thus was able to undergo the typical transitions associated with a serine proteinase inhibitor (as opposed to non-specific proteolysis). Note that trace amounts of RNC were co-purified with released AAT from rabbit reticulocyte lysate reactions. (c) AAT RNCs made using a non-stop linear DNA construct, before and after purification using a sucrose cushion. RNCs following treatment with RNase A release the full-length nascent chain with the expected molecular weight of 45 kDa (i.e., a band shift down of ~ 20kDa corresponds to the loss of the nascent chain-bound tRNA moiety). (d) Released AAT with a signal sequence forms an RNC during biosynthesis starting from mRNA in an uncoupled, translation-only reaction (reactions were not quenched with cycloheximide). This suggests that RNCs are not an artefact of cycloheximide treatment, and that stalling can also occur in the absence of microsomes. (e) Shows PNGaseF treatment of AAT targeted to microsomes. A collection of faint bands higher in molecular weight relative to both the RNCs and released nascent chains was observed, and indicative of glycosylation. PNGase F treatment under denaturing conditions dissociates the RNCs, and resolves all translated species into a single band consistent with full-length, deglycosylated AAT. (f) (left) A 60-minute biosynthesis time course for wild-type AAT in aurintricarboxylic acid-synchronised reactions, which were quenched with cycloheximide. Highlighted in the box the peak formation of RNCs, with the asterisk highlighting the approximate time at which RNC formation is at its highest. (right) As described in left, but shows Z AAT.

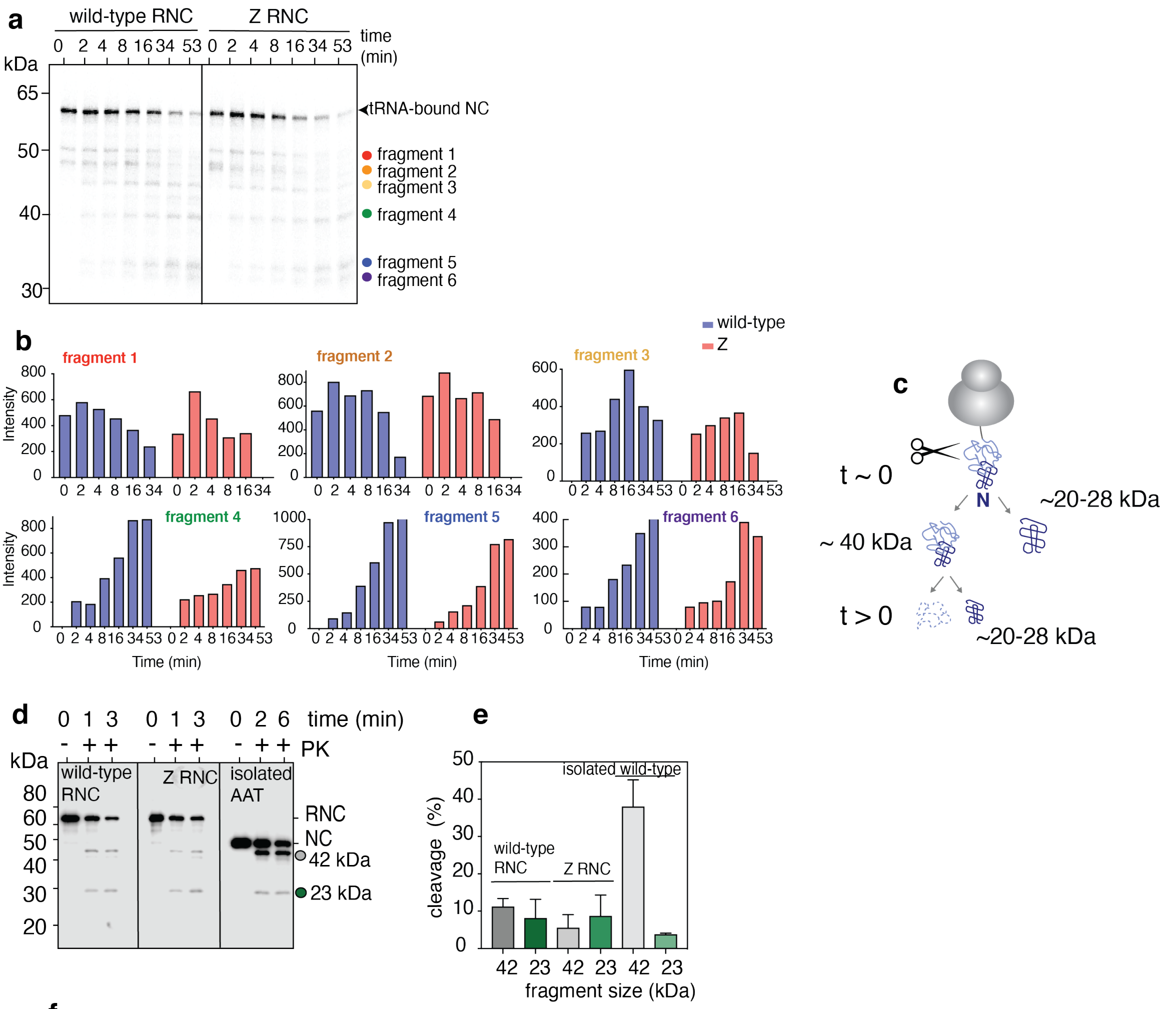

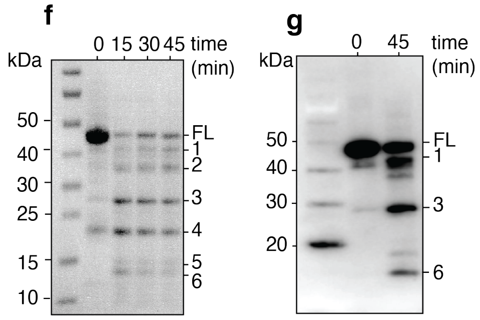

h

band	end sequence	predicted cleavage %	MW (Da)
1	LKLSK$_{343}$<br>PPEVK$_{365}$<br>QNTK$_{380}$	66.9%<br>**71.3%**<br>2.8%	38637<br>42184<br>44023
2	VKFN$_{367}$	71%	42446
3	DLVKE$_{175}$<br>YIFFK$_{191}$<br>VTTVK$_{217}$	55.2%<br>**85%**<br>3.2%	20809<br>22752<br>25883
4	MMKR$_{223}$	59%	25520
5	MFLE$_{354}$	67%	41021
6	LKLV$_{127}$	66.9%	15278

Supplementary Figure 2: Characterisation co-translational protein folding on the ribosome. (a) Limited proteolysis over time of wild-type and Z AAT RNCs using proteinase K, highlighting both ribosome-bound and released fragments, as shown by the coloured spheres. (b) Densitometric analysis of the released fragments over time. The fragment species are derived from the species marked by coloured spheres in (a). (c) A Proteolytic cleavage model for AAT-RNCs derived from the analysis of (b). (d) Proteinase K proteolysis of wild-type and Z RNCs and released AAT as monitored by an anti-His western blot (n=3 biological repeats). (e) Graph showing densitometric analysis of proteinase K digested fragments measured for the RNCs (at 3 mins) and released AAT (averaged, 4 mins). The two N-terminal fragments released from the RNCs (indicated by the grey and green spheres) are of equal intensities (~ 8 % of total cleaved product), compared to released AAT, where the 42 kDa fragment is more dominant (~ 36 % of total cleaved product) (see d). Data are presented as mean values +/- SEM. The N-terminal fragments of equivalent sizes are thus released from both RNCs and released AAT but to differing extents (i.e., the 23 kDa fragment appears to be more protected in released AAT relative to the RNC). These results suggest that the ribosome-bound nascent chain shares some structural preferences with that observed in released, natively-folded AAT, but it is otherwise highly labile.

Protease cleavage likely occurs close to the ribosomal exit tunnel around the first protease-accessible residues (at least 35 amino acids from the C-terminus) resulting in the 42 kDa fragment. As shown in (b), this 42 kDa species decreases over time, with a concomitant appearance of the 23 kDa species. The further fragmentation of the 42 kDa species suggests that it is of limited stability. By contrast, the 42 kDa fragment released from released, natively folded AAT does not fragment further (see f, g). This is likely a result of proteinase K cleavage occurring in the reactive centre loop (RCL) which catalyses a conversion towards the RCL-cleaved AAT conformation; its resistance to further proteolysis is a result of its hyperstability[71]. This same conversion does not occur in the RNC because it is only partially-folded. (f) Partially-denaturing PAGE with Coomassie-staining showing limited proteolysis of released AAT using proteinase K (8ng/μl), and identifying bands (as detected in (a)). (FL = full-length AAT) (g) As shown in (f), but detection of N-terminal fragments using an anti-His western blot. (h) Table shows the sequence identity of each of the bands/fragments excised from (f) as determined using mass spectrometry, in addition to the predicted cleavage at each site (in bold) as determined using EXPASY's PeptideCutter (chymotrypsin-model).

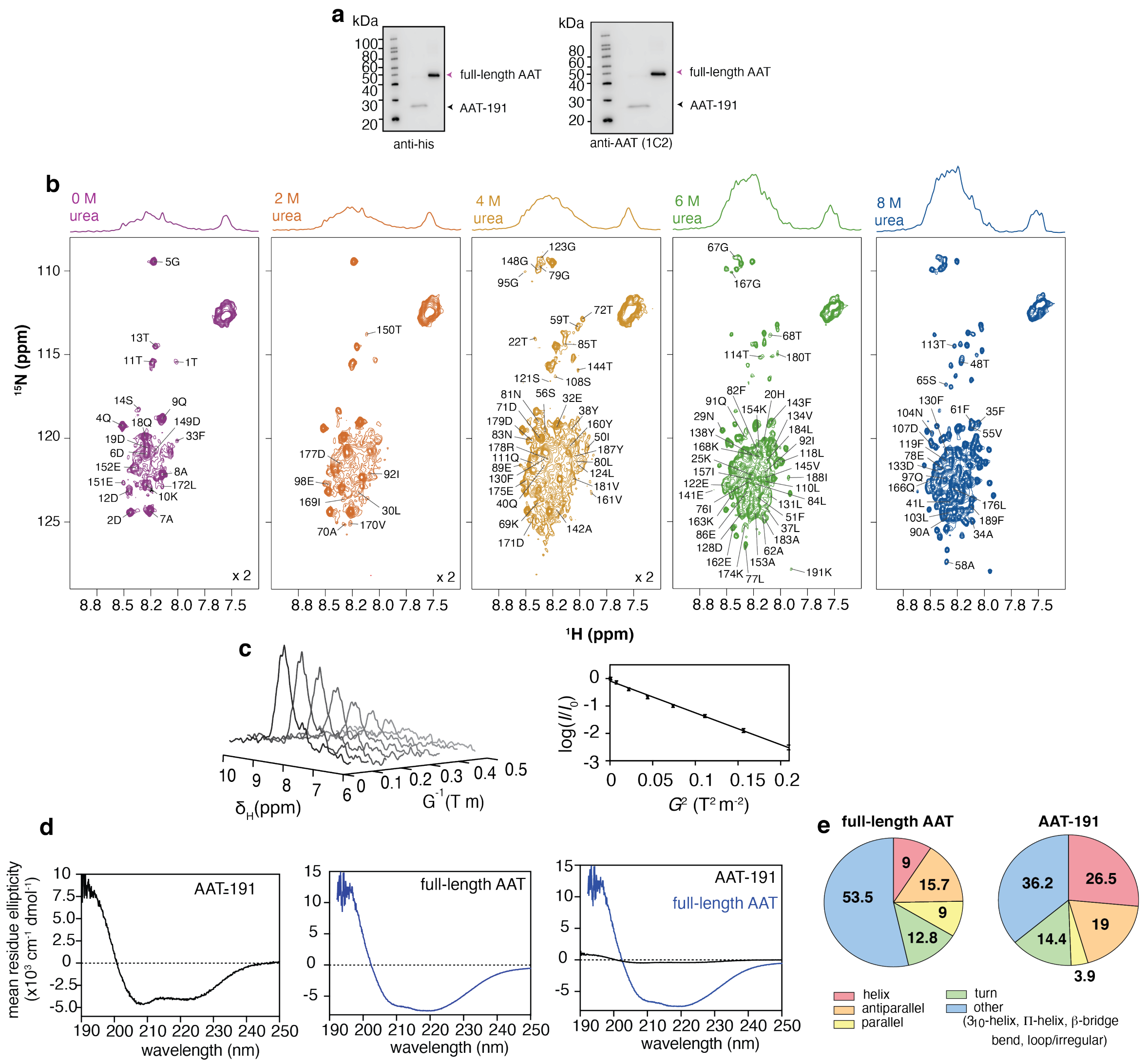

Supplementary Figure 3: Biochemical, biophysical, and NMR analysis of AAT 1-191. (a) (left) An anti-His Western blot of full-length, released AAT (10 pmol) and released 1-191 (380 pmol). (right) as in (a), using 1C2 anti-AAT antibody. (b) (upper)1D and (lower) 2D ^{1}H,^{15}N-SOFAST HMQC spectra of AAT-191 in progressively increased concentrations of urea, recorded at 25˚C and at a ^{1}H frequency of 800-MHz, Resonances are labelled with their assignment at the urea concentration in which they become observable. The signal threshold in 0 to 2M urea is increased (x2) relative to 6M and 8M urea due to weak signals. (c) (left) Translational diffusion measurements of AAT-191 under native conditions using an 8-point ^{1}H,^{15}N-XSTE experiment with a diffusion delay of 100 ms. (right) The natural logarithm of integrals of the amide region of the spectra were plotted as a function of the squared gradient strength to calculate the diffusion coefficient using the Stejskal-Tanner equation[72]. (d) Far-UV CD spectra of AAT-191, full-length AAT, and an overlay of AAT-191 and full-length AAT. All spectra were recorded at 25°C. (e) Estimation of secondary structure using the BestSel algorithm[73,74] applied to (left) full-length AAT (analysis range 200-250 nm) and (right) AAT-191 (analysis range 190-250 nm).

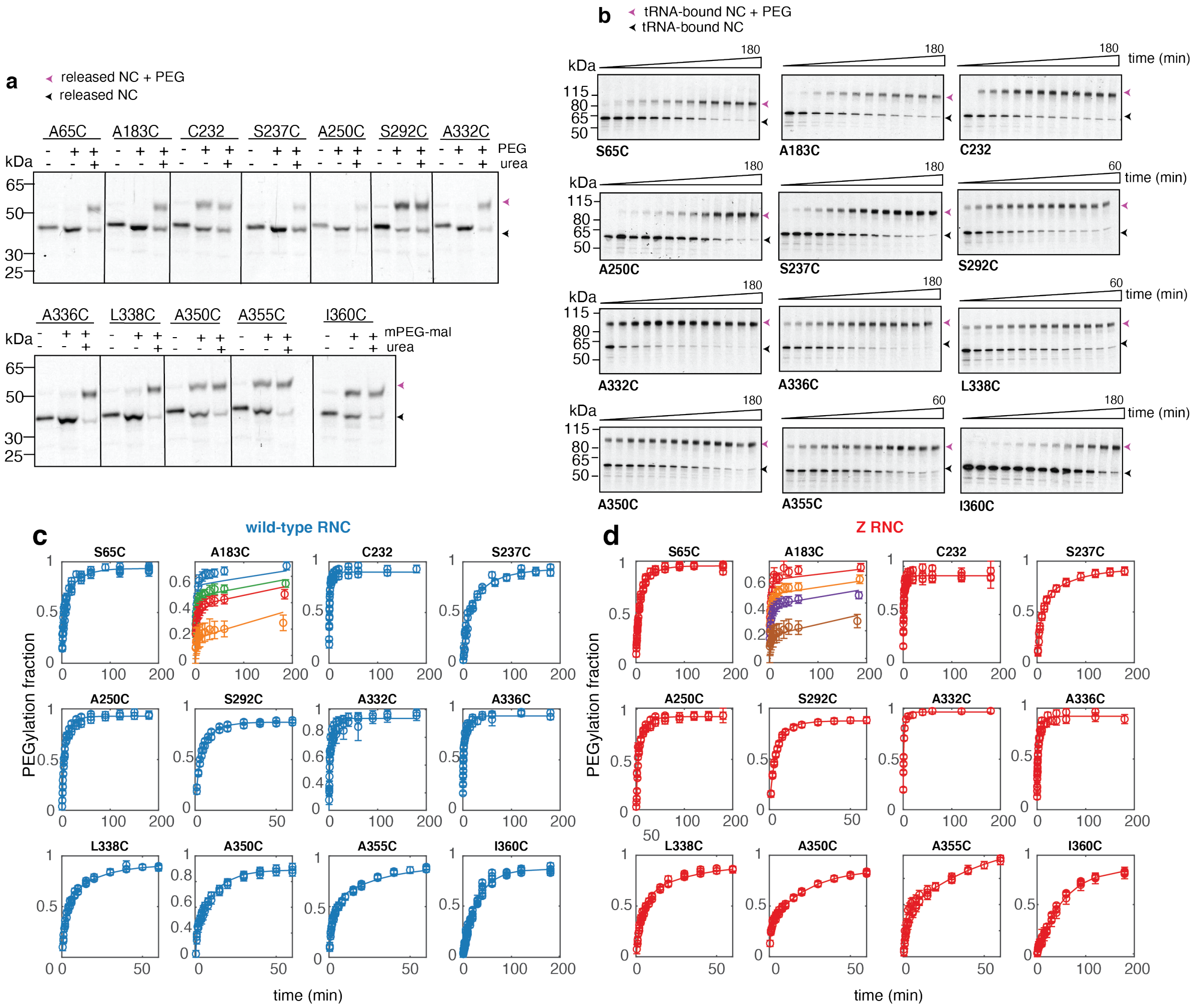

Supplementary Figure 4: PEGylation of released AAT and AAT RNCs. (a) Shows released AAT cysteine variants in rabbit reticulocyte lysate after 1 hour of folding and a 1-hour PEGylation reaction, as shown using partially-denaturing PAGE and ^{35}S methionine detection. Samples are in the absence and presence of 4.8 M urea. (b) PEGylation of wild-type RNCs without an RNC incubation time (see text), as monitored over time (0 to 180 minutes) using partially-denaturing PAGE and ^{35}S methionine detection. In these assays, it was found that approximately 65% of the RNCs remained intact after 22.5 hours at 25°C (see Supplementary Fig. 5c). This suggests that despite the integrity of the RNCs being compromised through nascent chain release or potential RNC self-association, selectively monitoring the tRNA-bound nascent chain (i.e., an RNC) enables us to report on the behaviour of ribosome-bound nascent chains. (c) Global fits of the extent of PEGylation for wild-type RNCs using a kinetic model for RNC behaviour (see also Supplementary Fig. 5a). The RNCs have 'zero incubation time', apart from 183C, which shows a range of incubation times (see also Supplementary Fig. 5b)). The kinetic model is derived from a numerical solution, the basis of which comes from experiments performed on 183C RNCs which were incubated at various times (see also Supplementary Fig. 5b and Methods) (n=5 biological repeats). The change in the extent of PEGylation was not due to cysteine oxidation over time (oxidation $t_{1/2} > 24$ h) nor related to PEG degradation ($t_{1/2} > 30$ h, as measured by NMR, data not shown). (d) As shown in (c), but for Z RNCs. All data are presented as mean values +/- SEM.

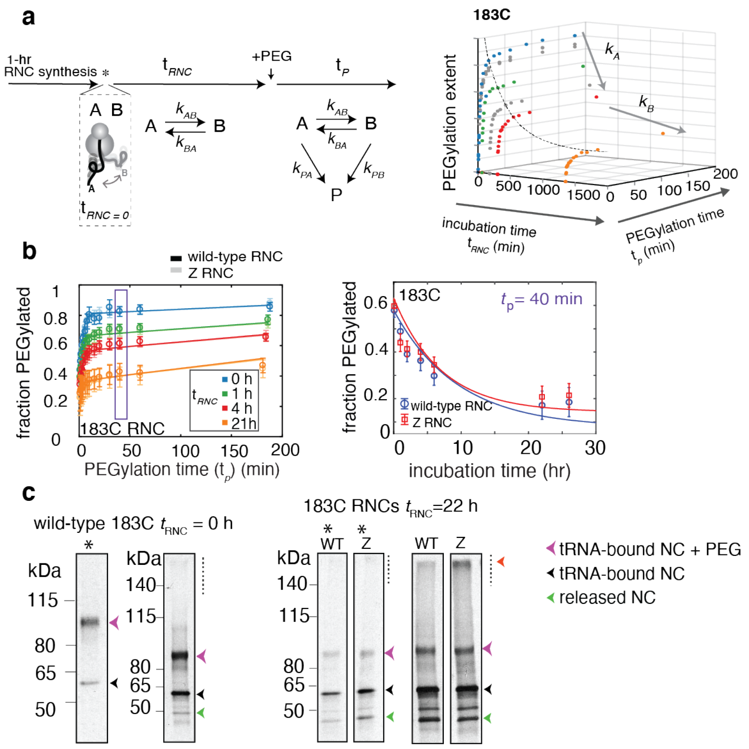

Supplementary Figure 5: Deriving a kinetic model for RNC PEGylation (a) (left) A kinetic model of RNC behaviour as measured from PEGylation experiments. (right) 3D representation of the PEGylation of 183C RNCs showing how the extent of PEGylation changes as a function of both incubation time and PEGylation reaction time. (b) (left) Change in PEGylation extent for 183C over incubation time. (n= 4 biological repeats). (right) The extent of PEGylation (after a 40 min PEGylation reaction) as a function of incubation time, as derived from (left). (c) Over-exposed, partially-denaturing gels showing PEGylation of wild-type and Z RNCs at incubation time = 0 and RNC incubation time = 22 h, with detection using ^{35}S methionine. Highlighted are high molecular weight (HMW) species (smear, dashed line), and an orange arrowhead denoting the top of the running gel. Also shown (*) is a gel of wild-type RNCs with no RNC incubation time using a standard exposure. Data in (c) and (d) are presented as mean values +/- SEM.

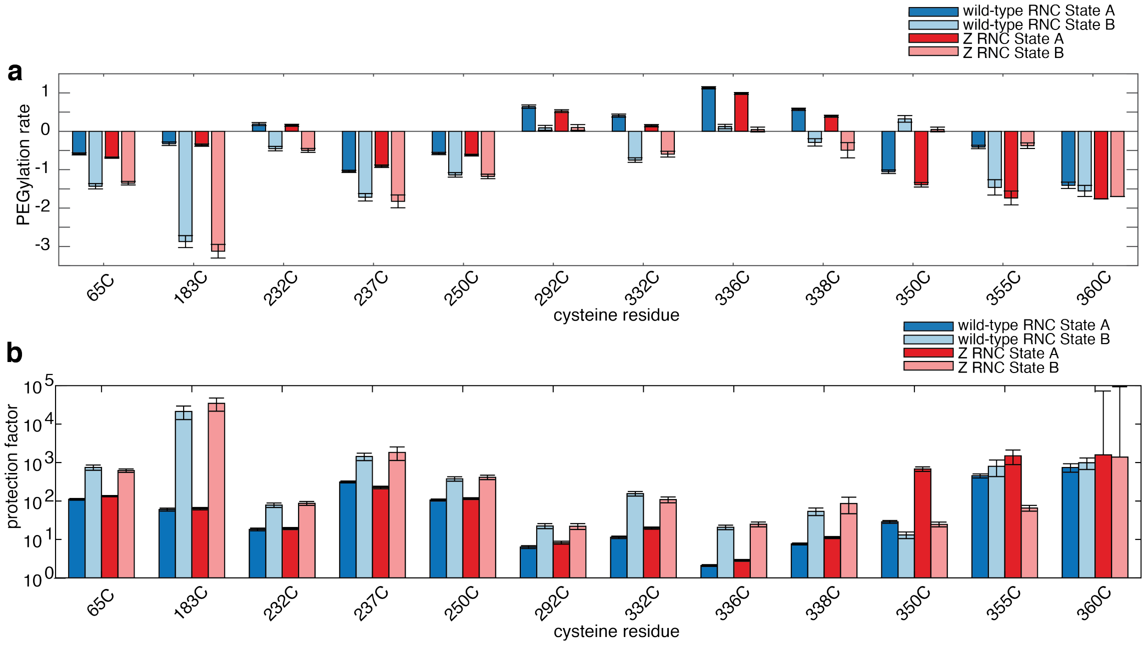

Supplementary Figure 6: PEGylation of wild-type and Z RNCs. (a) Shows PEGylation rates (expressed as log_{10} values) obtained for each cysteine residue in the co-translational folding intermediates for wild-type and Z RNCs (n=5 biological repeats). (b) As shown in (a), but expressed as protection factors (using PEGylation of Cys 747 in natively-unfolded FLN5 Y719E as a model for a fully solvent exposed cysteine residue (see text)). Data are presented as mean values +/- SEM.

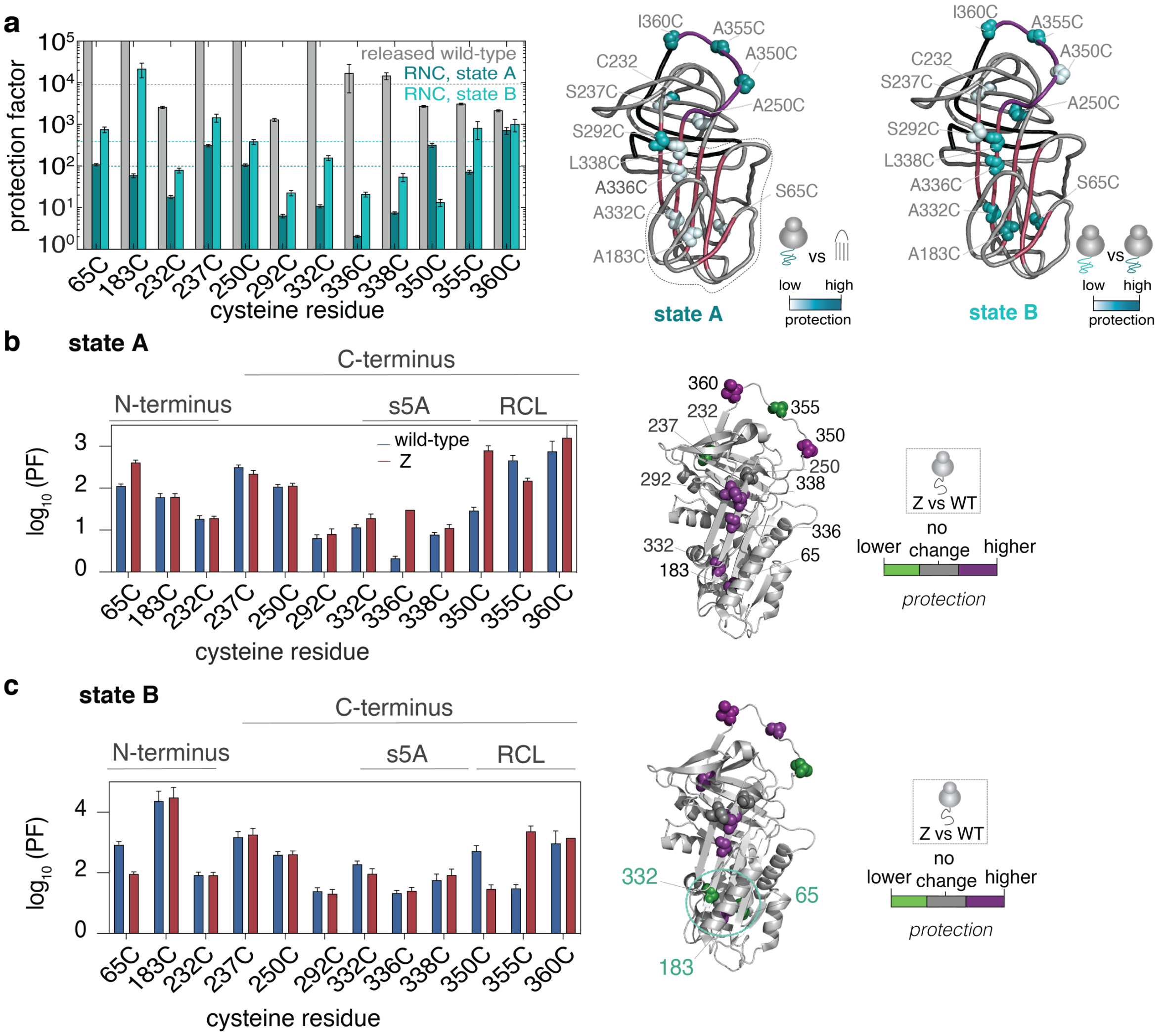

Supplementary Figure 7: RNC PEGylation and comparative analysis of states A and B in wild-type and Z RNCs. (a) (left) Protection factors of released AAT and wild-type RNC states A and B. The dashed lines represent the average protection factor values for released wild-type, and wild-type RNC states A and B. (right) Structural depictions of wild-type RNCs as derived from protection factors. RNC State A was derived from a comparison made with released, natively folded wild-type AAT, and RNC state B was derived from a comparison made with RNC state A (from left). Highlighted in dashed lines is the N-terminal fragment, 1-191 (n= 5 biological repeats). (b) (left) Relative difference in protection in the Z co-translational folding intermediate ('state A') compared to that of wild-type, as mapped onto AAT's native structure (this is as shown in Fig. 6a) (right). (n= 5 biological repeats). (c) As for (b) but shows RNC 'state B'. Wild-type and Z RNC showed similarities in state B, but with Z also showing a less protected N-terminus (cyan, circled). State B forms over time likely as a result of higher-order assembly or aggregation of an intact RNC. In wild-type RNCs, State A has protection factor values of ~ 10-100 and typically less protected relative to the misfolding-prone state B (PFs ~100-1000). The initial populations of states A and B in the wild-type RNC were 58 ± 2 % and 42 ± 2 %, respectively, suggesting that wild-type RNCs form a co-translational folding intermediate (state A) with a misfolding/aggregation potential (state B). As applied to Z RNCs, the same PEGylation analysis revealed similarities to wild-type, including similar initial populations of state A (63% ± 2 %) and state B (37± 2 %). (n= 5 biological repeats). Data are presented as mean values +/- SEM in (a) to (c).

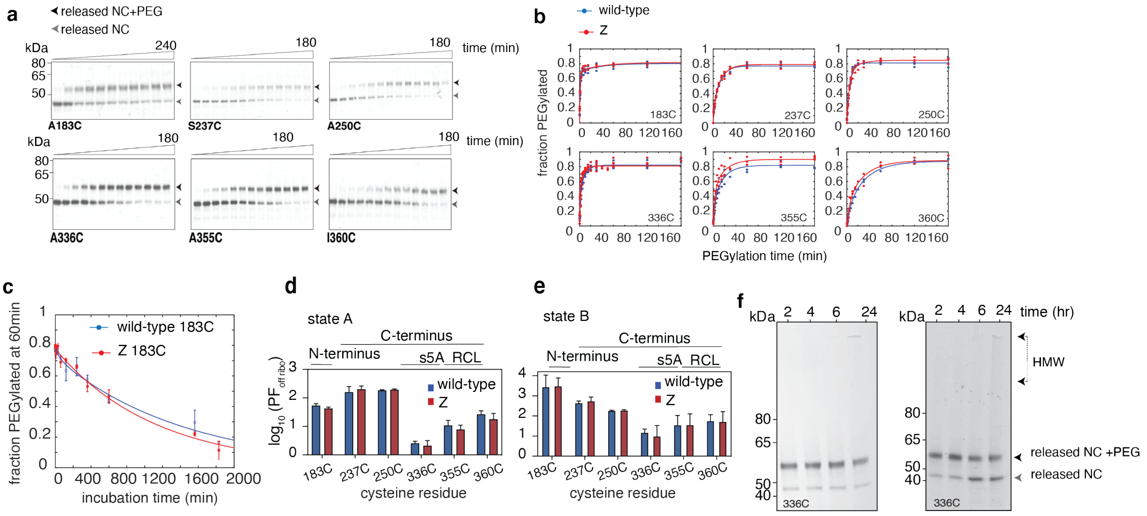

Supplementary Figure 8: AAT's post-translational folding intermediate monitored by PEGylation. (a) The extent of PEGylation for RNAse A-released, wild-type nascent chains and incubated in 3M urea and 1mM mPEG-mal at 25°C for 180 or 240 minutes. (b) Densitometric analysis of gels shown in (a) fitted with a numerical solution following a model similar to that used for the RNCs (see Methods). (c) The extent of PEGylation (after a 60 min PEGylation reaction) as a function of nascent chain incubation time, which shows the existence of two released nascent chain states, A and B (a similar analysis as performed for the RNCs, see Supplementary Fig.5). We did not focus on state B because of its likely involvement in polymerisation, a separate process (same rationale as used for the RNCs). (n=4 biological repeats) (d) Protection factors for state A for the post-translational intermediate of released wild-type and Z AAT. (n=4 biological repeats) (e) Protection factors for state B for the post-translational intermediate of released wild-type and Z AAT. (n=4 biological repeats). (f) PEGylation gel of 3M-urea denatured, released wild-type 336C of two different repeats (left) 4-hour incubation (right) 24-hour incubation, highlighting the presence of the PEGylated species (+ PEG) and the high molecular weight (HMW) species. Data are presented as mean values +/- SEM in (c) to (e).

Figure 9

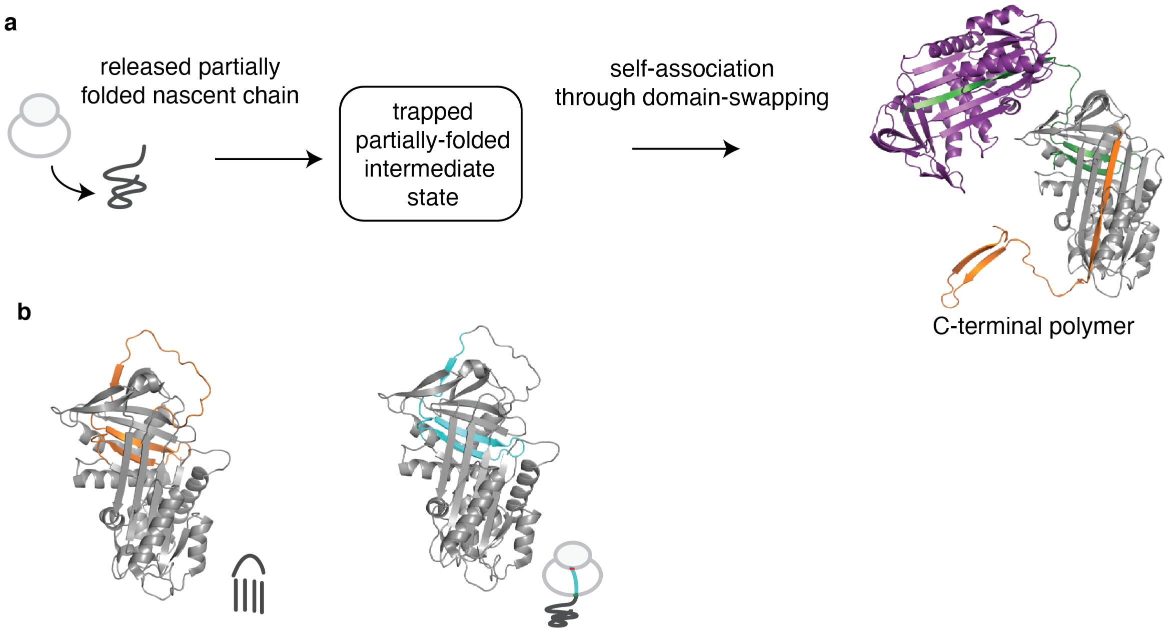

Supplementary Figure 9: A C-terminal polymerisation model for AAT during biosynthesis via a domain-swap. (a) A proposed mechanism for C-terminal polymerisation of AAT, as determined from a trimer structure of AAT (PDB ID: 3T1P)[34]. (b) (left) AAT monomeric structure with the C-terminal region (orange) that is implicated in the C-terminal domain swapping mechanism shown in (a). (right) AAT with the C-terminal region that is occluded in the tunnel in a ribosome translating full-length AAT. Following nascent chain release, the occluded C-terminal region is inserted into AAT's core to complete its native structure. It is anticipated that the Z mutation delays the insertion of this region, thus promoting a persistent partially-folded intermediate that is more vulnerable to a C-terminal domain-swapping event resulting in polymerisation (See also Fig. 8).

a

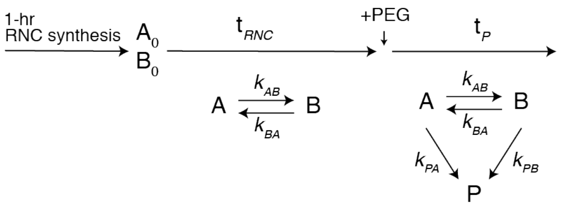

b

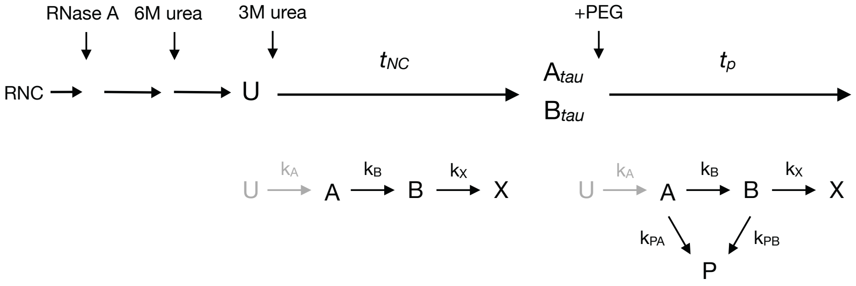

Supplementary Figure 10: Reaction schemes used to model and fit PEGylation kinetics data. (a) Shows the reaction scheme for RNCs. Immediately following synthesis $t_{RNC} = 0$, the RNCs exist in two states, A_0 and B_0. There is a reversible transition of state A to B (with rates k_{AB} and k_{BA}) within the RNC co-translational intermediate ensemble. Following the addition of PEG (t_p), k_{PA} and k_{PB} are distinct PEGylation rates for states A and B to form the PEGylated species (P). (b) Shows the reaction scheme for the post-translational intermediate. Following release, unfolding and refolding into 3M urea ($t_{NC} = 0$), the NC can transition from state A to state B (with rate k_B), which then undergoes a slow transition towards a polymerised state, X (with rate k_X). Following the addition of PEG ($t_p = 0$), the populations of A (A_{tau}) and B (B_{tau}) can be PEGylated (P) with rates of k_{PA} and k_{PB}. For both (a) and (b) please also refer to the Methods for additional details.
