# Supplementary material for: Nascent chains can form co-translational folding intermediates that promote post-translational folding outcomes in a disease-causing protein
Source: Nat Commun. 2021 Nov 8;12:6447. doi: 10.1038/s41467-021-26531-1 (PMC8576036; doi:10.1038/s41467-021-26531-1)
Supplement: Supplementary file 2 — Reporting Summary [file 41467_2021_26531_MOESM2_ESM.pdf]

nature portfolio

Corresponding author(s): John Christodoulou & Lisa Cabrita

Last updated by author(s): Sep 10, 2021

Reporting Summary

Nature Portfolio wishes to improve the reproducibility of the work that we publish. This form provides structure for consistency and transparency in reporting. For further information on Nature Portfolio policies, see our Editorial Policies and the Editorial Policy Checklist.

Please do not complete any field with "not applicable" or n/a. Refer to the help text for what text to use if an item is not relevant to your study.
For final submission: please carefully check your responses for accuracy; you will not be able to make changes later.

Statistics

For all statistical analyses, confirm that the following items are present in the figure legend, table legend, main text, or Methods section.

n/a	Confirmed	
☐	☒	The exact sample size (*n*) for each experimental group/condition, given as a discrete number and unit of measurement
☐	☒	A statement on whether measurements were taken from distinct samples or whether the same sample was measured repeatedly
☒	☐	The statistical test(s) used AND whether they are one- or two-sided<br>*Only common tests should be described solely by name; describe more complex techniques in the Methods section.*
☒	☐	A description of all covariates tested
☐	☒	A description of any assumptions or corrections, such as tests of normality and adjustment for multiple comparisons
☐	☒	A full description of the statistical parameters including central tendency (e.g. means) or other basic estimates (e.g. regression coefficient) AND variation (e.g. standard deviation) or associated estimates of uncertainty (e.g. confidence intervals)
☒	☐	For null hypothesis testing, the test statistic (e.g. *F*, *t*, *r*) with confidence intervals, effect sizes, degrees of freedom and *P* value noted<br>*Give P values as exact values whenever suitable.*
☒	☐	For Bayesian analysis, information on the choice of priors and Markov chain Monte Carlo settings
☒	☐	For hierarchical and complex designs, identification of the appropriate level for tests and full reporting of outcomes
☒	☐	Estimates of effect sizes (e.g. Cohen's *d*, Pearson's *r*), indicating how they were calculated

Our web collection on statistics for biologists contains articles on many of the points above.

Software and code

Policy information about availability of computer code

Data collection	- NMR data were collected using Bruker TopSpin software (v3.5pl5)<br>- Circular dichroism data were collected using Chirascan software (Applied Photophysics, v.4.7.0.194) and secondary structure determination was undertaken using BestSel, available at: https://bestsel.elte.hu/<br>- Western blot chemiluminescence was collected using ImageStudio (v.4.0)
Data analysis	- Image J (v 1.52K) and Fityx (v 1.3.1) were used for densitometric analysis of autoradiography films and western blots. As needed, Adobe Photoshop CC (v 19) was used to adjust the contrast across (entire) images of scanned autoradiography films.<br>-MatLab (vR2019b or R2017a) was used for mathematical modeling and fitting of protein biosynthesis, folding and PEGylation kinetics.<br>-NMR data processing and analysis was undertaken using NMRPipe and CCPN Analysis 2.4.2

For manuscripts utilizing custom algorithms or software that are central to the research but not yet described in published literature, software must be made available to editors and reviewers. We strongly encourage code deposition in a community repository (e.g. GitHub). See the Nature Portfolio guidelines for submitting code & software for further information.

Data

Policy information about availability of data

All manuscripts must include a data availability statement. This statement should provide the following information, where applicable:
- Accession codes, unique identifiers, or web links for publicly available datasets
- A description of any restrictions on data availability
- For clinical datasets or third party data, please ensure that the statement adheres to our policy

The authors declare that all data supporting the findings of this study are available within the article and its Supplementary Information Files.

Field-specific reporting

Please select the one below that is the best fit for your research. If you are not sure, read the appropriate sections before making your selection.

☒ Life sciences ☐ Behavioural & social sciences ☐ Ecological, evolutionary & environmental sciences

Life sciences study design

All studies must disclose on these points even when the disclosure is negative.

Sample size	No sample size was predetermined. Sample sizes were chosen according those generally accepted in the co-translational protein folding field.
Data exclusions	No data were excluded from analyses.
Replication	Each experiment was reproduced at least 3 times (often more). The results were replicated consistently across multiple experiments and generated similar results.
Randomization	Randomization was not applicable for the experiments in this study. These were in vitro experiments designed to be systematic and performed using well-controlled conditions.
Blinding	Blinding was not applicable for the experiments undertaken in this study.

Reporting for specific materials, systems and methods

We require information from authors about some types of materials, experimental systems and methods used in many studies. Here, indicate whether each material, system or method listed is relevant to your study. If you are not sure if a list item applies to your research, read the appropriate section before selecting a response.

Materials & experimental systems

n/a	Involved in the study	
☐	☒	Antibodies
☒	☐	Eukaryotic cell lines
☒	☐	Palaeontology and archaeology
☒	☐	Animals and other organisms
☒	☐	Human research participants
☒	☐	Clinical data
☐	☒	Dual use research of concern

Methods

n/a	Involved in the study	
☒	☐	ChIP-seq
☒	☐	Flow cytometry
☒	☐	MRI-based neuroimaging

Antibodies

Antibodies used	Penta-His HRP (Qiagen, catalogue number 34460), Anti-antitrypsin mouse monoclonal antibody 1C2 (Sigma, catalogue number
Validation	Validation for antibodies used as supplied by the manufacturer's specifications provided in the product information:<br>Penta-His HRP: Detection of N-, C- and internal 6xHis tags; Antibody isotype (Mouse 1gG1); Negligible cross-reactivity with crude E.coli, yeast, mammalian or insect cell lysates<br>1C2: https://www.sigmaaldrich.com/deepweb/assets/sigmaaldrich/product/documents/319/013/sab4200198dat.pdf

Dual use research of concern

Policy information about dual use research of concern

Hazards

Could the accidental, deliberate or reckless misuse of agents or technologies generated in the work, or the application of information presented in the manuscript, pose a threat to:

No	Yes	
☒	☐	Public health
☒	☐	National security
☒	☐	Crops and/or livestock
☒	☐	Ecosystems
☒	☐	Any other significant area

Public health

Experiments of concern

Does the work involve any of these experiments of concern:

No	Yes	
☒	☐	Demonstrate how to render a vaccine ineffective
☒	☐	Confer resistance to therapeutically useful antibiotics or antiviral agents
☒	☐	Enhance the virulence of a pathogen or render a nonpathogen virulent
☒	☐	Increase transmissibility of a pathogen
☒	☐	Alter the host range of a pathogen
☒	☐	Enable evasion of diagnostic/detection modalities
☒	☐	Enable the weaponization of a biological agent or toxin
☒	☐	Any other potentially harmful combination of experiments and agents

This checklist template is licensed under a Creative Commons Attribution 4.0 International License, which permits use, sharing, adaptation, distribution and reproduction in any medium or format, as long as you give appropriate credit to the original author(s) and the source, provide a link to the Creative Commons license, and indicate if changes were made. The images or other third party material in this article are included in the article's Creative Commons license, unless indicated otherwise in a credit line to the material. If material is not included in the article's Creative Commons license and your intended use is not permitted by statutory regulation or exceeds the permitted use, you will need to obtain permission directly from the copyright holder. To view a copy of this license, visit http://creativecommons.org/licenses/by/4.0/
